# *Helicobacter pylori*-induced aberrant methylation of ID4 mediated by DNMT3B drives gastric cancer progression via DEC1-SHH signaling pathway

**DOI:** 10.1038/s41419-025-08042-9

**Published:** 2025-10-07

**Authors:** Muhua Luan, Wenshuai Zhu, Zhaotian Feng, Fubo Jing, Yuanxin Xing, Xiaoli Ma, Yunshan Wang, Bin Ning, Yanfei Jia

**Affiliations:** 1 Research Center of Basic Medicine, Jinan Central Hospital, Shandong University, Jinan, People’s Republic of China; 2 Research Center of Basic Medicine, Central Hospital Affiliated to Shandong First Medical University, Jinan, People’s Republic of China; 3 Department of Medical Laboratory, Shandong Second Medical University, Weifang, People’s Republic of China; 4 Central Hospital Affiliated to Shandong First Medical University, Shandong First Medical University, Jinan, People’s Republic of China

**Keywords:** Epigenetics, Tumour biomarkers, Gastrointestinal diseases

## Abstract

*Helicobacter pylori* (*H. pylori*) infection mediates activation of oncogenes and inhibition of tumor suppressor genes through aberrant DNA methylation, which is the predominant risk factor for gastric tumorigenesis. Here, by integrating transcriptome and epigenetic multi-omics analyses of gastric tissues and mouse models, we identified that inhibitor of differentiation 4 (ID4) was downregulated in *H. pylori*-infected gastric tissues and associated with prognosis of gastric cancer (GC). *H. pylori* infection remarkably increased the methylation level of the ID4 promoter region in the GC patients and mouse models. ID4 served as a tumor suppressor gene in GC and was required for *H. pylori*-mediated tumorigenic activities in vitro cellular and in vivo tumor-bearing mouse models. Moreover, *H. pylori* infection induced DNMT3B upregulation through recruiting KLF5 to its promoter and further promoted ID4 DNA methylation modification. Notably, ID4 formed heterodimers with the basic HLH transcription factors DEC1 to inhibit its transcriptional activity; therefore, downregulation of ID4 promoted SHH/GLI1 signaling through a DEC1 dependent transcriptional modulation. Collectively, our findings indicate *H. pylori* infection depends on DNMT3B to induce ID4 DNA methylation and ID4 promoter hypermethylation status is a potential biomarker to identify GC. Loss of ID4 could be a key component of *H. pylori*-mediated gastric tumorigenesis through dysregulation of DEC1/SHH/GLI1 axis, which provides potential therapeutic targets in GC.

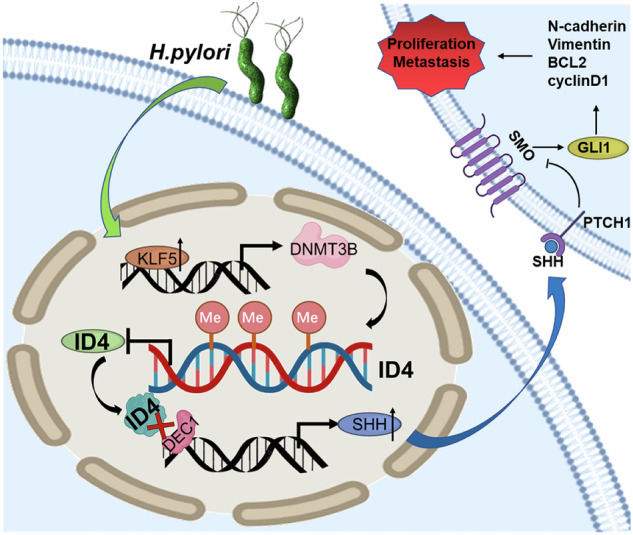

## Introduction

Gastric cancer (GC) is the most common and severe type of gastrointestinal cancers and the fourth largest cancer-related mortality globally [1]. Therefore, identifying signaling pathways and molecules relevant to the biology of GC is crucial for improving therapeutic strategies in the fight against GC*. Helicobacter pylori* (*H. pylori*) is a spiral-shaped gram-negative bacteria that colonizes the human stomach and is the predominant risk factor for gastric tumorigenesis [2, 3]. The World Health Organization (WHO) has classified infection with *H. pylori* as a class 1 carcinogen [4]. *H. pylori* infection mediated activation of oncogenes and inhibition of tumor suppressor genes through epigenetic mechanisms, promoting the development and progression of GC [5, 6]. Thus, delineating the molecular mechanisms and functional significance of *H. pylori* epigenetically deregulated genes in gastric carcinogenesis holds great value in the development of novel key gene targets for monitoring GC progression.

Inhibitor of differentiation 4 (ID4) belongs to the family of classic basic helix-loop-helix (bHLH) transcription factors but lacks the basic DNA-binding domain [7]. Id proteins were known to function by forming heterodimers with bHLH transcription factors, preventing them from binding to E-box DNA motifs and inhibiting transcriptional activity. Therefore, they are considered dominant negative regulators of differentiation pathways [8]. In tumorigenesis, ID1-ID3 proteins upregulate oncogenes such as *MYC* or *RAS* and downregulate tumor suppressor genes such as *TP53* [9]. However, ID4 appears to primarily act as a tumor suppressor in most cancers as opposed to ID1-3 [10]. Several studies have shown that methylation of the ID4 promoter is associated with tumor progression and poor prognosis. In breast cancer, methylation-related silencing of ID4 is a poor prognostic indicator, and demethylation is proposed as a means of overcoming ID4 repression [11]. Furthermore, the reduction in ID4 expression in prostate cancer has also been attributed to promoter hypermethylation [12]. In GC, a previous study found that hypermethylation of ID4 promoter occurs in GC, which is correlated with decreased levels of ID4 expression [13]. However, the critical mediators participating in DNA methylation of ID4 in GC have not been elucidated.

Even though DNA methylation is a well-characterized epigenetic modification in cancer, many new discoveries have been made over the past decade, including enzymes and functional contexts that involved [14]. DNA methylation is catalyzed by DNA methyltransferase family (DNMTs), which transfers a methyl group from S-Adenosylmethionine (SAM) to the fifth carbon of the cytosine residue, forming 5mC [15]. Three canonical DNMTs isoforms have been identified in humans, including DNMT1, DNMT3A and DNMT3B [16]. Since DNMTs are frequently overexpressed in tumors, DNMTs are excellent targets for treating cancer [17]. *H. pylori-*induced inflammation is essential for aberrant promoter DNA methylation, which is a hallmark of GC [18]. A deeper understanding of the molecular mechanisms responsible for the induction of DNA methylation by *H. pylori* and specifically DNMTs in this process is crucial for dissecting GC progression mechanisms and is helpful for developing rationalized combinatorial therapeutic approaches.

The Hedgehog (Hh) signaling pathway is of pivotal importance in epithelial development and differentiation, homeostasis and neoplastic transformation of the stomach [19]. Aberrant activation of Hh signaling is closely associated with various pathological consequences including GC [20]. Sonic Hedgehog (SHH), one of the Hh family members, has been demonstrated to be fundamental to the initiation of gastritis in response to *H. pylori* infection [21]. In fact, SHH/GLI signaling and its target genes control the major hallmarks of cancer, including proliferation, survival, metastasis and self-renewal, making it a promising target for cancer therapies [22].

Here, integrated transcriptome and epigenetic multi-omics analyses of gastric tissues and mouse models indicated that ID4 was downregulated in GC. Moreover, *H. pylori* infection inhibited ID4 expression via DNMT3B-mediated DNA methylation modification in GC by employing cell lines, mouse models and human samples. As an inhibitor of DNA-binding protein, ID4 interacted with transcription factor DEC1 to inhibit its transcriptional activity, thereby blocking GC malignant processes by inhibiting downstream targets SHH/GLI1 signaling pathway. Notably, we confirmed that the SHH inhibitor, vismodegib significantly enhanced the inhibitory effect of ID4 on GC tumor growth and metastasis. Overall, we provided new insights to *H. pylori* -mediated ID4 DNA methylation and elucidated a novel molecular mechanism underlying gastric carcinogenesis.

## Results

### ID4 is the key tumor suppressor gene in GC and associated with *H. pylori* infection and DNA methylation

**Fig. 1:**
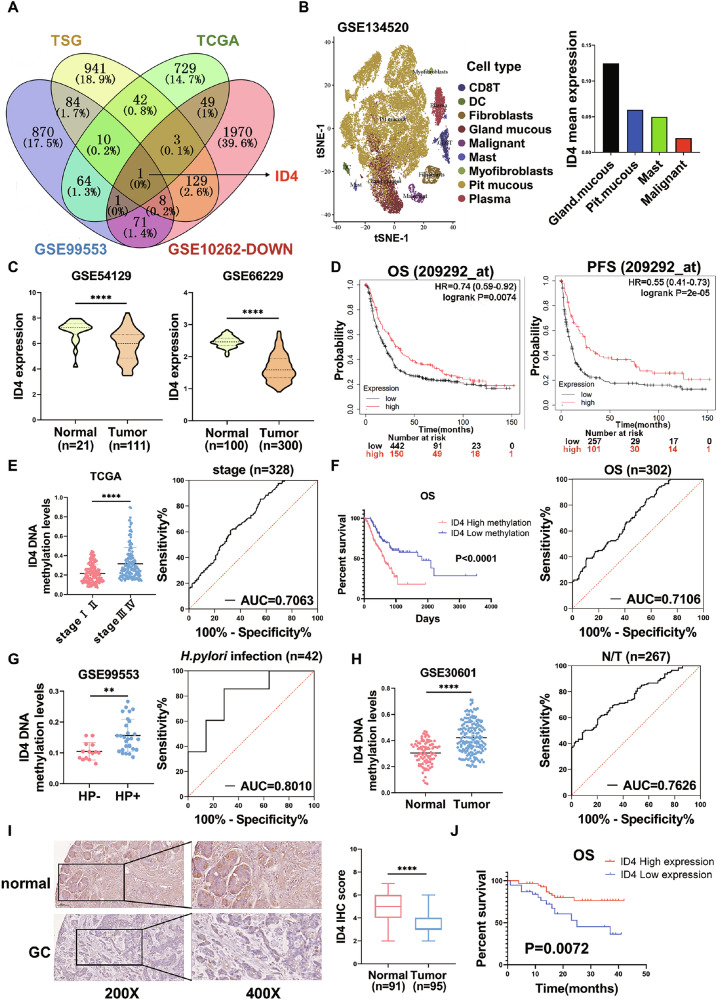
ID4 is the key tumor suppressor gene in GC and associated with H. pylori infection and DNA methylation. **A** Analyses of four datasets to identify tumor suppressor genes that are associated with *H. pylori* infection and DNA methylation. **B** The distribution of the ID4 in based on the single-cell RNA sequencing profile of the GSE134520 dataset. **C** The mRNA expression of ID4 is downregulated in tumor tissues compared to normal tissues from the GEO database (GSE54129, GSE66229). **D** Kaplan–Meier plotter analysis revealed that patients with lower ID4 expression have worse overall survival (OS) and progression-free survival (PFS) compared to patients with higher ID4 expression. ROC curve analysis revealed that ID4 methylation has clinical diagnostic efficacy in stage (**E**), prognosis (**F**), *H. pylori* infection status (**G**) and N/T tissues (**H**). **I** IHC score and IHC staining of ID4 in adjacent normal tissues (n = 91) and GC tissues (n = 95). **J** Overall survival was analyzed using Kaplan–Meier curves (log-rank test) in a GC cohort of 96 patients. *P < 0.05, **P < 0.01, ***P < 0.001, ****P < 0.0001.

To identify key tumor suppressor genes that are abnormally expressed in gastric cancer and exclusively associated with *H. pylori* infection and DNA methylation, we analyzed the TSGene database, the TCGA gastric cancer cohort, GSE99553 dataset (Methylation profiling data from gastric cancer cases with or without *H. pylori* infection), and GSE10262 dataset (Expression data from *H. pylori*-infected mice). We identified one overlapping gene ID4 (Fig. 1A). We further investigated the distribution of the ID4 in based on the single-cell RNA sequencing profile of the GSE134520 dataset. A total of 9 cell types were identified (Fig. 1B). ID4 were mainly enriched in gastric grand mucous cells. In contrast, the lower expression levels of ID4 in malignant cells. These results were further verified in two validation cohorts (GSE54129 and GSE66229). The mRNA expression of ID4 was also decreased in GC tissues (*P* < 0.0001, Fig. 1C). Subsequently, we determined the prognostic value of ID4 using the publicly accessible Kaplan–Meier plotter online platform and found that patients with GC having a low ID4 expression exhibited shorter overall survival (OS, *P* < 0.01) and progression-free survival (PFS, *P* < 0.0001, Fig. 1D). To further analyze the diagnostic efficacy of ID4 methylation, we selected 328 samples for gastric cancer stage (AUC = 0.7063) and 301 samples for prognosis (AUC = 0.7106) from TCGA database. Furthermore, we analyzed GSE99553 database to validate the correlation between ID4 methylation and *H. pylori* infection status (n = 42, AUC = 0.8010). We also analyzed GSE30601 database to indicate the diagnostic value of ID4 methylation in normal and tumor tissues (n = 267, AUC = 0.7626). These results suggested that ID4 methylation has clinical diagnostic efficacy (Fig. 1E–H). We further validated ID4 expression associated with our own clinical GC tissue cohort. IHC analysis showed that the expression of ID4 in the GC tissue samples was lower than that in the normal tissues (*P* < 0.0001, Fig. 1I). Analysis of the relationship between ID4 expression and clinicopathological parameters showed that ID4 expression was associated with *H. pylori* infection (*p* = 0.008), degree of differentiation (*p* = 0.004), T classification (*p* = 0.025), N classification (*p* = 0.036), and clinical staging (*p* = 0.001) (Table S1). In addition, survival curves showed that patients with low ID4 protein expression in our cohort displayed adverse OS (*P* < 0.01, Fig. 1J). Moreover, we further detected the protein levels of ID4 in 12 paired GC tissue samples by western blot, which was consistent with the above results (*P* < 0.0001, Fig. S1A). These results suggest that ID4 acts as a tumor suppressor in GC and is associated with *H. pylori* infection.

### *H. pylori* infection inhibits ID4 expression by inducing methylation modification of the ID4 promoter region

**Fig. 2:**
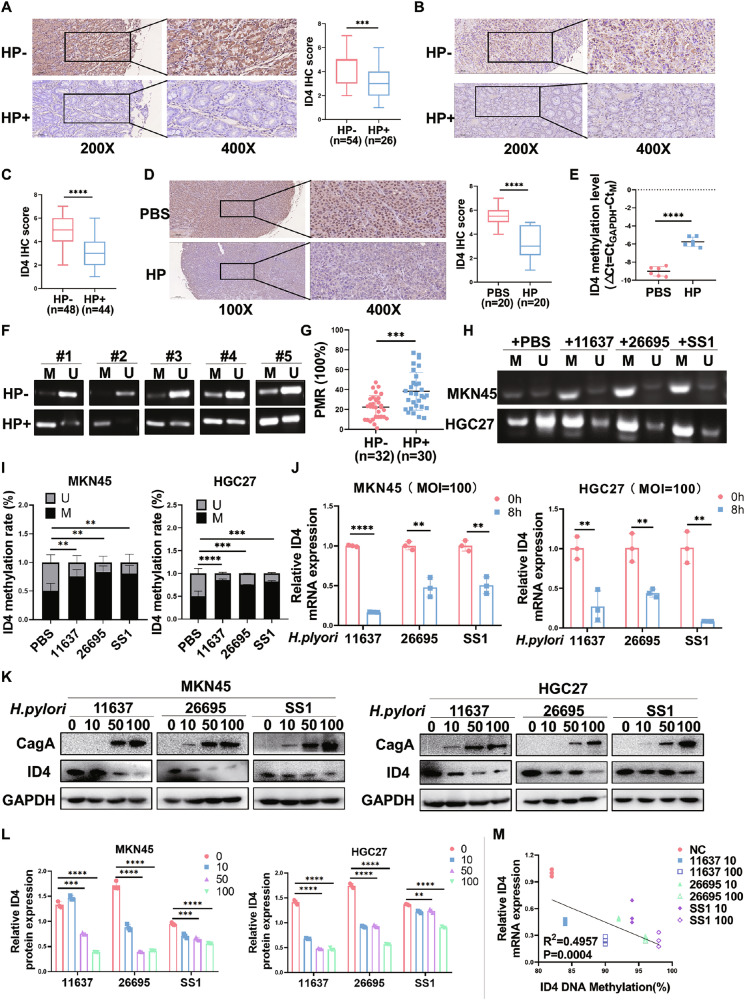
*H. pylori* infection inhibits ID4 expression by inducing methylation modification of the ID4 promoter region. **A**–**C** IHC staining and IHC score of ID4 in *H. pylori*-negative, *H. pylori*-positive patients with AG (**A**) and GC (**B**). **D** IHC staining and IHC score of ID4 in the PBS group (n = 20), *H. pylori* (HP, SS1 strain)-infected (n = 20) mice. **E** MSP analysis of the promoter methylation level of ID4 in the PBS group (n = 6) and HP (SS1 strain)-infected (n = 6) mice. **F**, **G** MSP and MethyLight analysis of the promoter methylation level of ID4 in *H. pylori*-negative (HP-; n = 32) and *H. pylori*-positive (HP + ; n = 30) GC samples. **H**, **I** MSP analysis of the promoter methylation level of ID4 after *H. pylori* infection (11637, 26695 and SS1; 8 h) in MKN45 and HGC27 cells. **J** RT-qPCR detection of ID4 mRNA expression after infection of the GC cell lines, MKN45 and HGC27 with *H. pylori* 11637, 26695 and SS1 for 8 h (MOI = 100). **K**, **L** Western blot analysis of ID4 and CagA protein expression in MKN45 and HGC27 cells infected with *H. pylori* 11637, 26695 and SS1 for 8 h. **M** BSP and RT-qPCR analysis revealed that ID4 methylation level is significantly correlated with its mRNA expression. *P < 0.05, **P < 0.01, ***P < 0.001, ****P < 0.0001.

A study has reported that *H. pylori* infection induces a chronic inflammatory environment with histopathological progression from chronic atrophic gastritis to intestinal metaplasia, dysplasia, and ultimately gastric cancer [23]. First, we validated ID4 expression levels with a clinical atrophic gastritis (AG) tissue cohort. IHC analysis of the human AG tissue cohort showed high levels of ID4 protein in *H. pylori*-negative (HP-) tissue samples compared to those in *H. pylori*-positive (HP + ) samples (*P* < 0.001, Fig. 2A). Notably, IHC analysis of the human GC tissue cohort obtained a similar result (*P* < 0.0001, Fig. 2B–C). Therefore, we performed in vivo and in vitro experiments to further demonstrate whether *H. pylori* infection plays an important role in regulating ID4 expression. We infected C57BL/6 mice with *H. pylori* strain SS1 via gavage for 4 months. IHC analysis revealed that the *H. pylori*-infected group had significantly downregulated ID4 expression in the gastric mucosa compared to the PBS group (*P* < 0.0001, Fig. 2D). Western blot analysis showed that the expression of ID4 was reduced in *H. pylori*-infected mice, consistent with the above results (Fig. S1B–C).

Numerous studies have shown that chronic inflammation of the gastric epithelium due to *H. pylori* infection induces aberrant DNA methylation, which disrupts downstream signaling [24]. Promoter methylation is known to be an important epigenetic mechanism associated with tumor suppressor genes silencing in cancer. We also analyzed the correlation between DNA methylation levels and ID4 expression using the online website cBioPortal and the results revealed a significant negative correlation between ID4 expression and its DNA methylation level in GC tissues (*R* = −0.37, *P* < 0.001, Fig. S1D). Therefore, we hypothesized that *H. pylori* infection induces methylation modification of the ID4 promoter region. We first designed specific primers based on the CpG islands and subsequently performed MSP, which has been widely used for methylation detection. MSP analysis revealed that *H. pylori* infection significantly increased the methylation level of the ID4 promoter region in *H. pylori*-infected mice compared to the PBS group (*P* < 0.0001, Fig. 2E). Consistent with the above results, MSP analysis of *H. pylori*-negative (HP-) and *H. pylori*-positive (HP + ) GC samples from our cohort also demonstrated significantly increased methylation levels in the ID4 promoter region in HP + GC samples (*P* < 0.0001, Fig. 2F). We also designed a methylation specific probe for MethyLight assay to quantify the methylation level of ID4. We analyzed promoter methylation of ID4 quantitatively in the HP- (n = 32) and HP+ (n = 30) GC samples from our cohort using the MethyLight assay and the results showed a significant increase in methylation level (PMR value) in HP + GC samples, which is in accordance with our hypothesis (*P* < 0.001, Fig. 2G). To further validate the results in tissue samples, we infected MKN45 and HGC27 cells with *H. pylori* strains (11637, 26695 and SS1) for 8 h. MSP analysis demonstrated that *H. pylori* infection increased the methylation level of the ID4 promoter region in MKN45 and HGC27 cells (Fig. 2H, I). RT-qPCR results showed that ID4 mRNA expression was significantly reduced in GC cell lines after 8 h of infection with *H. pylori* strains (Fig. 2J). Similarly, western blot analysis showed that *H. pylori* infection significantly downregulated the ID4 protein levels in an MOI-dependent manner in the human GC cell lines for 8 h, and the expression level was lowest at an MOI of 100 (Fig. 2K, L). Furthermore, to further analyze the correlation between DNA methylation and gene expression in a dose-dependent manner, we selected MOI of 10 and 100 to quantitatively analyze the methylation sites and mRNA expression of ID4 by BSP and RT-qPCR. The results showed that *H. pylori* infection increased the methylation level of the ID4 and revealed a significant negative correlation between ID4 methylation level and its mRNA expression level in an MOI-dependent manner (Fig. 2M and Fig. S1E). Overall, *H. pylori* infection induced methylation modification of the ID4 promoter region and inhibited ID4 expression in human GC cell lines and clinical tissue samples.

#### ID4 exerts tumor-suppressive effects in GC cells and organoids

**Fig. 3:**
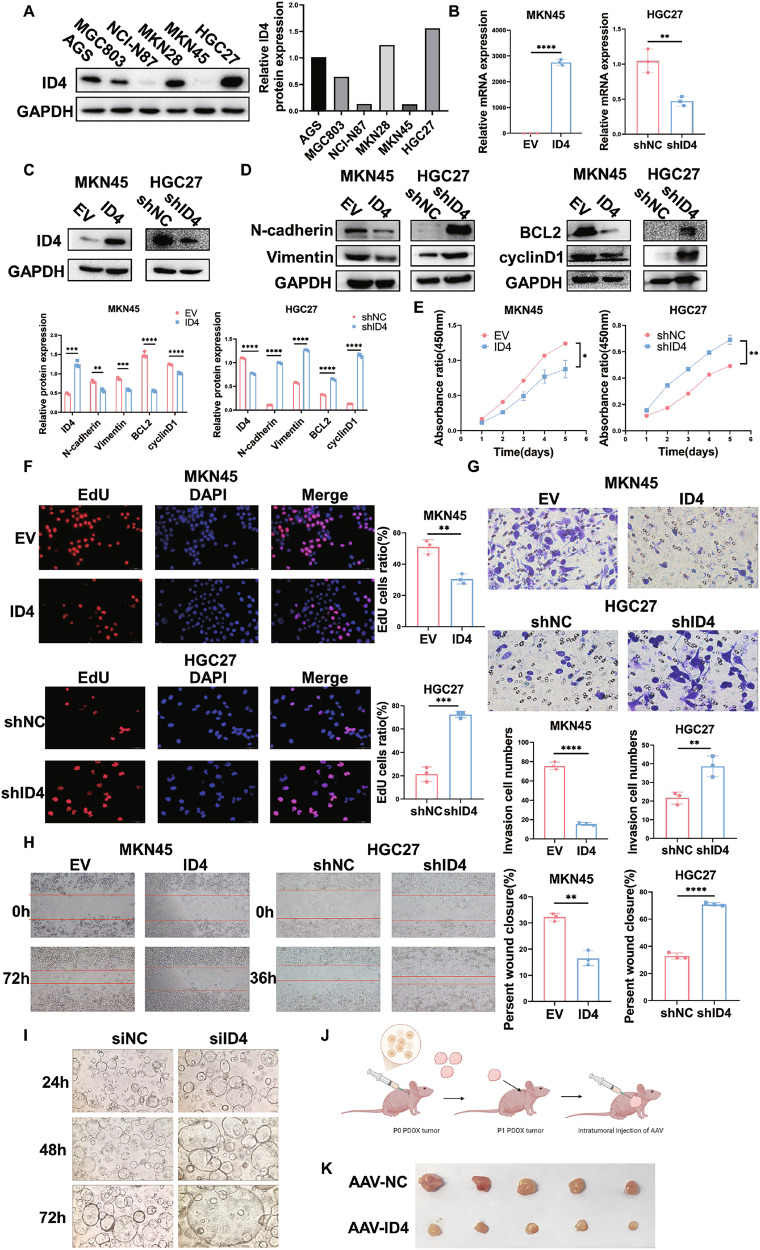
ID4 exerts tumor-suppressive effects in GC cells and organoids. **A** Western blot analysis of the protein expression levels of ID4 in multiple GC cell lines. RT-qPCR (**B**) and western blot analysis (**C**) of the transfection efficiency of overexpression of ID4 in MKN45 cells or shRNA-mediated ID4 repression in HGC27 cells. **D** Overexpression or knockdown of ID4 influenced the protein expression of mesenchymal markers (N-cadherin and vimentin), BCL2 and cyclinD1 in western blot assay. CCK-8 assays (**E**) and EdU assays (**F**) were performed to monitor cell proliferation abilities in MKN45 and HGC27 cells. Transwell assays (**G**) and wound healing assays (**H**) of MKN45 and HGC27 cells were conduced to measure the migration capabilities. Representative images and quantification data are shown. All data are presented as the mean ± standard deviation of three independent experiments. **I** ID4 knockdown promoted the growth of organoids. **J** PDOX model (n = 5 mice per group) was established using GC patient-derived organoids, followed by intratumoral injection of AAV- ID4 into the tumors. *P < 0.05, **P < 0.01, ***P < 0.001, ****P < 0.0001.

Given the fact that ID4 expression is decreased in GC tissues and functions as a tumor suppressor in GC, we speculated that ID4 might inhibit GC cells proliferation and migration in vitro. We detected the protein levels of ID4 in GC cell lines (AGS, MGC803, NCI-N87, MKN28, MKN45 and HGC27) by western blot and found that ID4 possessed high expression in HGC27 but low expression in MKN45 (Fig. 3A). Therefore, to confirm the role of ID4 in GC, we established ID4 overexpression cell model in MKN45 cells and ID4 knockdown cell model in HGC27 cells. The transfection efficiency was determined by RT-qPCR and western blot (Fig. 3B, C). Next, we performed western blotting to detect the expression of EMT and cell proliferation regulatory proteins in GC cell lines. Our data showed that ID4 overexpression significantly decreased the expression of N-cadherin, vimentin, BCL2 and cyclinD1 in MKN45 cells. On the other hand, ID4 knockdown in HGC27 cells led to an opposite result (Fig. 3D). The cell proliferative capabilities of GC cells were assessed by CCK-8 and EdU assays. As expected, overexpression of ID4 remarkably exhibited inhibitory effects on cell viability and proliferative potential (Fig. 3E, F). In addition, transwell and wound healing assays showed that upregulation of ID4 dramatically inhibited the migration capabilities of MKN45 cells (Fig. 3G, H). Likewise, knockdown of ID4 facilitated proliferative (Fig. 3E, F) and migration capabilities (Fig. 3G, H) compared to the control group.

Organoids are three-dimensional tissue-resembling cellular clusters derived from tissues that mimic tumor characteristics and could be used to develop novel anti-cancer treatments. By transfecting siRNA targeting ID4 into organoids, we discovered that ID4 knockdown significantly promoted the growth of organoids (Fig. 3I). Then, we established an organoid-based xenograft (PDOX) model. In this model, tumors derived from established organoids were implanted into nude mice, serving as a robust platform for the in vivo validation of the organoid system. Initially, we successfully established organoids in nude mice and harvested P0 generation tumor tissue. Subsequently, P1 generation tumor transplantation experiments were conducted in similar nude mice. Tumor establishment was followed by ID4-AAV approximately two weeks later to assess its impact on tumorigenesis. The results showed that ID4 significantly inhibited the growth of organoids, which further validate the therapeutic effect of targeting ID4 (Fig. 3J).

### *H. pylori* infection downregulates ID4 expression via KLF5-DNMT3B-mediated DNA methylation modifications

**Fig. 4:**
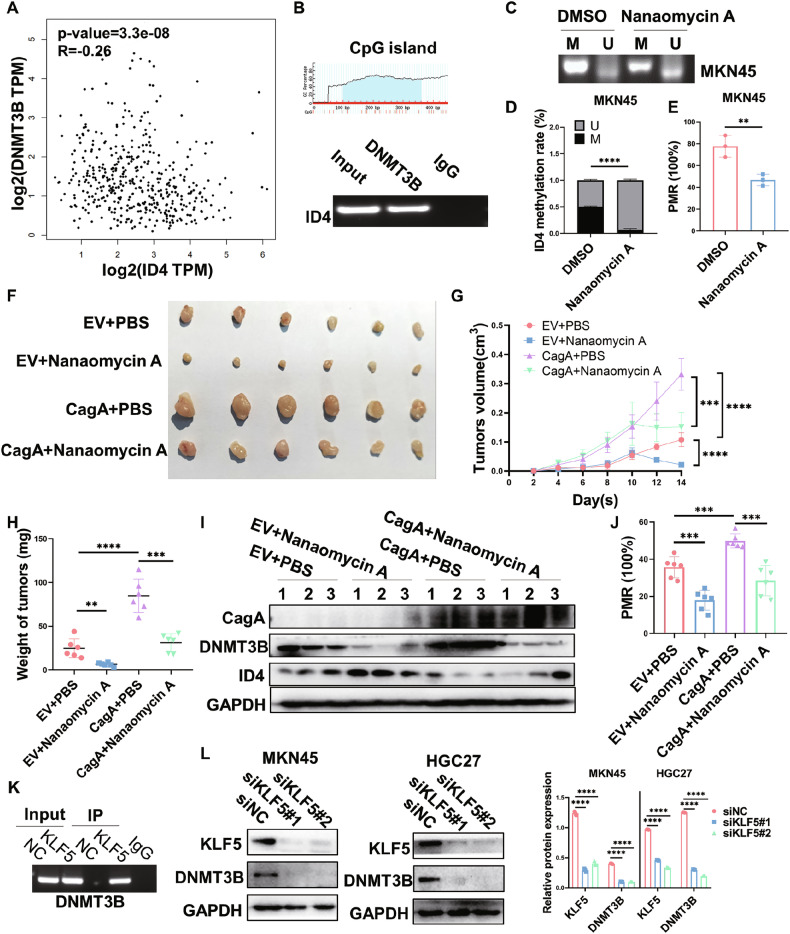
*H. pylori* infection downregulates ID4 expression via KLF5-DNMT3B-mediated DNA methylation modifications. **A** GEPIA analysis revealed that ID4 expression was significantly correlated with DNMT3B in patients with GC (Spearman method, *R* = −0.26, *P*<0.001). **B** ChIP was performed to detect the DNMT3B binding region of the ID4 promoter CpG island. **C–E** Promoter methylation level of ID4 in MKN45 cells treated with Nanaomycin A (500 nM) through MSP (**C**) and MethyLight (**E**). **F** The effects of CagA combined with Nanaomycin A treatment on tumor growth of MKN45 cells (n = 6 mice per group). Tumor growth curve (**G**) and tumor weight (**H**) were measured. Western blot (**I**) and MethyLight (**J**) analysis of ID4 protein expression and promoter methylation level in xenograft tumors**. K** ChIP was performed to detect the KLF5 binding region of the DNMT3B promoter. **L** Western blot analysis of KLF5 and DNMT3B protein expression in MKN45 and HGC27 cells transfected with control siRNA and KLF5 siRNA. *P < 0.05, **P < 0.01, ***P < 0.001, ****P < 0.0001.

DNA methylation is catalyzed by the DNA methyltransferase family (DNMTs) [25]. Overexpression of DNMTs results in abnormal DNA methylation patterns [26]. In this research, we analyzed the correlation between DNMTs (DNMT1, DNMT3A, DNMT3B) and ID4 expression in GC to search for methylation enzymes that regulate ID4 expression using the GEPIA online database. Only DNMT3B showed the most significant correlation with ID4 in terms of expression (*R* = −0.26, *P* < 0.001, Fig. 4A, Fig. S2A). Furthermore, RT-qPCR analysis demonstrated a significant upregulation of DNMT3B in GC cells after *H. pylori* (11637, 26695, SS1) infection (Fig. S2B). Consistently, DNMT3B protein expression was also increased after *H. pylori* infection (Fig. S2C). Due to DNMT3B regulates gene expression by methylation of the CpG islands in the promoter region of target genes, we hypothesized that DNMT3B could regulate the transcription level of ID4 through methylation of the CpG island region in the ID4 promoter. Using online software, we designed one pair of ChIP primers for the CpG island region in the ID4 promoter. Then, these were used to perform PCR amplification on the DNA fragments that were precipitated using DNMT3B antibody or IgG from the cell lysate. The result showed that the amount of product amplified was higher in the DNMT3B group than in the IgG group (Fig. 4B). Nanaomycin A is the first DNMT3B-selective inhibitor identified to induce genomic demethylation [27]. Next, we treated MKN45 and HGC27 cells with different concentrations of Nanaomycin A for 24 h to determine whether DNMT3B regulates the expression and methylation levels of ID4. Our results showed that the expression of ID4 was upregulated in a dose-dependent manner when Nanaomycin A was added to MKN45 and HGC27 cells (Fig. S2D). Furthermore, knocked down DNMT3B achieved a similar result (Fig. S2E). Notably, MSP and MethyLight analysis demonstrated that treated MKN45 cells with Nanaomycin A (500 nM) resulted in reduced methylation in the ID4 promoter region (Fig. 4C, E).

DNA hypomethylating drugs reprogram tumor cells to a normal-like state and sensitize the cells to chemotherapy and immunotherapy. This led to the pursuit of DNMT inhibitors for the treatment of cancer, and two DNMT inhibitors, decitabine (DAC) and azacitidine (AZA), have been used in the clinic [28]. DAC is known as the most extensively studied first-generation DNMT inhibitor which targets DNMT1, DNMT3A and DNMT3B. Our results showed that the expression of ID4 was upregulated in a dose-dependent manner when DAC was added to MKN45 cells (Fig. S2G). To further demonstrate the efficacy of ID4 and DAC, MKN45 tumor-bearing mice were intratumorally injected with DAC twice a week. The results demonstrated that DAC treatment exhibited potent antitumor activity, which further enhanced by ID4 (Fig. S2H–J). However, studies have found that these agents result in DNA damage and dose-limiting toxicities, thereby limiting their clinical application [29]. CagA, encoded by the cag-pathogenicity island (cag PAI) of *H. pylori*, has been recognized as a marker of the entire cag island [30]. To test whether CagA is linked to ID4 expression levels, we established a CagA overexpression cell model in MKN45 cells and western blot assay showed that CagA significantly decreased the expression of ID4 (Fig. S2F). Subsequently, we subcutaneously injected CagA-overexpressing and negative control MKN45 cells into BALB/c nude mice. To further demonstrate the efficacy of Nanaomycin A, MKN45 tumor-bearing mice were intratumorally injected with Nanaomycin A twice a week. The results demonstrated that Nanaomycin A treatment exhibited potent antitumor activity (Fig. 4F–H). Western blot and MethyLight analysis demonstrated that CagA could inhibit the expression levels of ID4 by inducing methylation modification of the ID4 promoter region, which could be reversed by Nanaomycin A treatment (Fig. 4I, J). These results provide compelling preclinical evidence that Nanaomycin A represents a promising therapeutic strategy for GC.

Krüppel-like factor 5 (KLF5), a member of a family of zinc-finger transcription factors, is a host factor that mediates oncogenic signaling pathways in the gastrointestinal tract [31]. The Signaling Pathways Project analysis revealed that KLF5 is the first transcription factor predicted to bind to the promoter region of DNMT3B in the human stomach (Fig. S3A). Previous research has shown that *H. pylori* infection promotes the expression of KLF5 in vitro and in vivo [32]. Our western blot analysis also obtained the same results in MKN45 and HGC27 cells infected with *H. pylori* (Fig. S3B). Some studies have also shown that *H. pylori* could aberrantly activate the Wnt/β-catenin signal and ectopically induce Wnt target genes including CDX1, thereby inducing KLF5 expression [33]. Additionally, elevated phosphorylation of the transcription factor STAT3 is a feature of GC, including *H. pylori*-infected tissues [34–36]. Jin et al. have suggested that STAT3 could transcriptionally regulate KLF5 expression [37]. Our western blot analysis showed that phosphorylation of STAT3 was increased after *H. pylori* infection, while knocking down STAT3 significantly decreased the expression of KLF5 (Fig. S3C-D). These results indicated that *H. pylori* induced KLF5 by elevating phosphorylation of STAT3. Using online software, we analyzed the DNMT3B promoter and identified possible KLF5 binding sites. Subsequently, we designed a paired primer and performed a ChIP assay, which revealed that KLF5 could directly bind to the promoter of DNMT3B to influence its expression (Fig. 4K). Furthermore, western blot assay showed that knocked down KLF5 significantly decreased the expression of DNMT3B (Fig. 4L). Collectively, these results suggested that *H. pylori* inhibited its expression by inducing the activation of KLF5-DNMT3B-mediated ID4 methylation modifications.

### ID4 interacts with DEC1 to co-regulate downstream signaling pathways

**Fig. 5:**
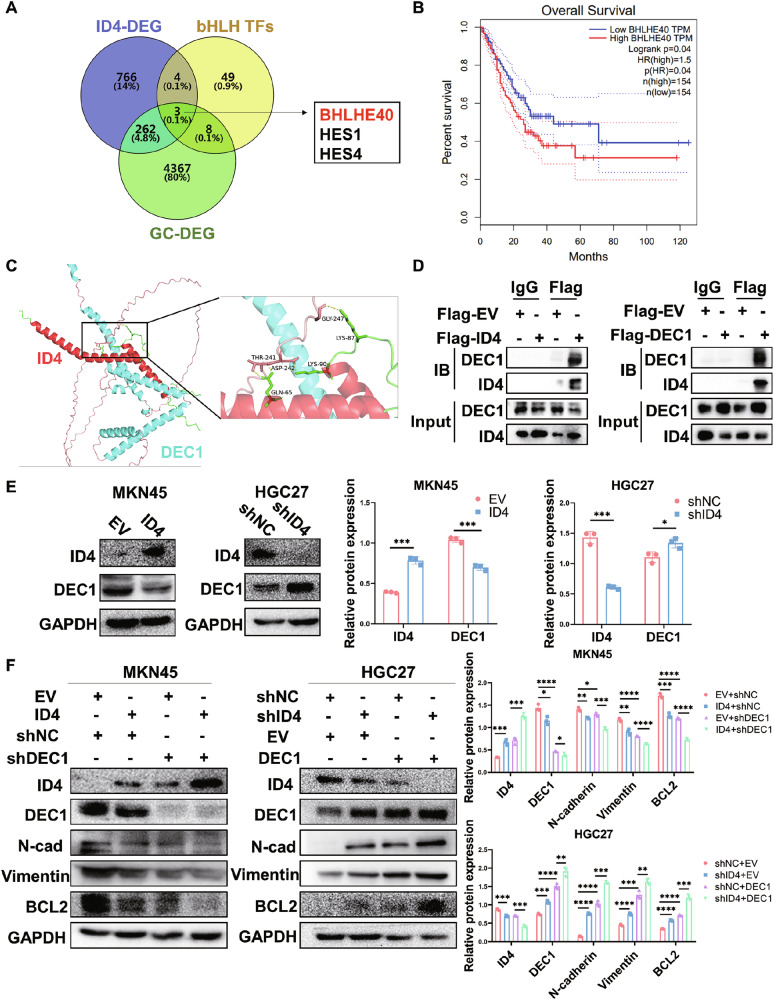
ID4 interacts with DEC1 to co-regulate downstream signaling pathways. **A** Venn diagram showed the overlap of differentially expressed bHLH TFs in GC and ID4 by RNA-seq. **B** GEPIA analysis showed that patients with higher BHLHE40 expression have worse OS compared to patients with lower BHLHE40 expression. **C** Molecular docking between ID4 and DEC1 using Alphafold 3. **D** Protein-protein interactions were determined by co-IP in HEK-293T cells. **E** Western blot analysis of DEC1 (BHLHE40) protein expression in ID4-overexpression and ID4-knockdown GC cells. **F** ID4 overexpression combined with DEC1 knockdown in MKN45 cells or ID4 knockdown combined with DEC1 overexpression in HGC27 cells influenced the protein expression of mesenchymal markers (N-cadherin and vimentin) and BCL2 in western blot assay. *P < 0.05, **P < 0.01, ***P < 0.001, ****P < 0.0001.

As ID4 has no DNA binding ability and usually interacts with the bHLH transcription factor family to inhibit downstream signaling pathways, we performed RNA sequencing in ID4 overexpression and control MKN45 cells and screened out the DEGs in GC from the TCGA database to identify ID4-bound transcription factors (TFs). From TFs that potentially interacted with ID4, three bHLH TFs were selected, of which only BHLHE40 (DEC1) correlates with poor prognosis in GC (Fig. 5A, B, Fig. S4A). Molecular docking prediction analysis of proteins using Alphafold 3 showed that ID4 interacted with DEC1 through three binding sites (Fig. 5C, Fig. S4B). Consistent with the above results, Co-IP analysis and immunofluorescence co-localization experiments further confirmed the protein-protein interactions between ID4 and DEC1 (Fig. 5D, Fig. S4C). Among three binding sites, Q65 and T241 have more hydrogen bonds and stronger bond energy. Therefore, to pinpoint the exact regions within the proteins that mediate this interaction, we designed ID4 mutant located in this site. Co-IP analysis revealed that ID4 mutant (Q65R) abolished the binding with DEC1, compared with ID4 wildtype (WT) (Fig. S4D).

In addition, western blot analysis showed that ID4 overexpression suppressed the expression levels of DEC1 (Fig. 5E). To further confirm the role of ID4 in combination with DEC1 in GC, we established a DEC1 knockdown cell model in MKN45 cells and a DEC1 overexpression cell model in HGC27 cells (Fig. S5A). Next, we silenced DEC1 in ID4-overexpressed MKN45 cells, and western blot analysis showed that silencing DEC1 further enhanced the EMT and cell proliferation regulatory protein inhibitory effects of ID4. On the other hand, overexpressed DEC1 in ID4-knockdown HGC27 cells led to opposite results (Fig. 5F). Based on these observations, we suggested that ID4 interacts with DEC1 protein to co-regulate downstream signaling pathways.

### Silencing DEC1 enhances the cell proliferation, migration and tumor growth inhibitory effects of ID4 in vitro and in vivo

**Fig. 6:**
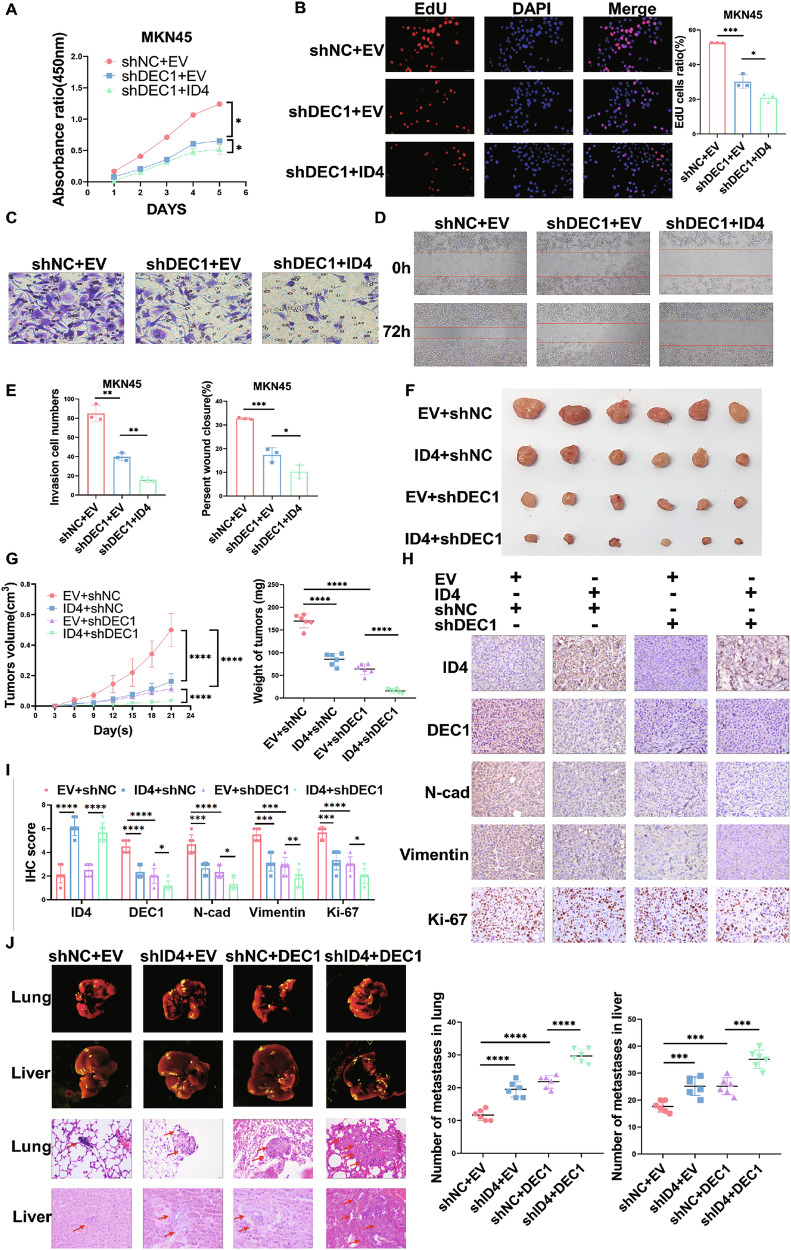
Silencing DEC1 enhances the cell proliferation, migration and tumor growth inhibitory effects of ID4 in vitro and in vivo. The effects of ID4 and DEC1 on proliferative ability of GC cells were measured by CCK-8 assays (**A**) and EdU assays (**B**). **C–E** The effects of ID4 and DEC1 on migration ability of GC cells were determined by transwell assays (**C**) and wound healing assays (**D**). **F**, **G** Subcutaneous tumor models were established using stable ID4-overexpressed and negative control MKN45 cells in combination with separately transfected shRNA-mediated DEC1 repression cells. Growth curves and tumor weights (**G**) were analyzed. Mass images of tumors collected from mice are shown (**F**). IHC staining (**H**) and IHC score (**I**) of protein expression levels of ID4, DEC1, EMT markers and Ki-67 in xenograft tumors. **J** Representative mass images of metastatic tumor nodules in the lung and liver of mice and representative H&E images of lung and liver samples from the indicated groups of nude mice. The number of metastasis tumor nodules in the lung and liver and statistically analyzed. *P < 0.05, **P < 0.01, ***P < 0.001, ****P < 0.0001.

To assess the influence of DEC1 on ID4-overexpression and ID4-knockdown GC cells, we performed CCK-8, EdU, transwell and wound healing assays. Our data indicated that silencing DEC1 could enhance the inhibitory effects of ID4 on the proliferation and migration abilities of MKN45 cells, whereas the results of overexpressed DEC1 in ID4-knockdown HGC27 cells were contrary to those described above (Fig. 6A–E, Fig. S5B–F). Subsequently, we performed animal experiments to validate the data obtained with cell lines. We subcutaneously injected stable ID4-overexpressed and negative control MKN45 cells in combination with separately transfected shRNA-mediated DEC1 repression cells into BALB/c nude mice. The tumors were harvested on the 21st day and further analyzed. We found that both ID4 overexpression and DEC1 knockdown inhibited tumor growth and decreased tumor weight. Additionally, knocked down DEC1 in ID4-overexpressed MKN45 cells further enhanced tumor suppression (*P* < 0.0001, Fig. 6F, G). Subsequently, EMT markers (N-cadherin and vimentin) and Ki-67 IHC staining again showed that inhibition of DEC1 could further impair the proliferation and migration of tumor cells in ID4-overexpressing MKN45 cells (Fig. 6H, I). Next, to clarify the effects of ID4 and DEC1 on the metastasis of GC, we injected stable ID4-knockdown and negative control HGC27 cells, as well as respectively transfected DEC1 overexpression cells, into the tail vein of 5-week-old BALB/c mice. The mice were sacrificed 4 weeks after injection and their lungs and livers were collected. We observed that inhibition of ID4 and activation of DEC1 promoted the metastasis of GC with an increase in liver and pulmonary metastasis. Moreover, overexpressed DEC1 in ID4-knockdown HGC27 cells further enhanced tumor metastasis and histopathological findings confirmed these results (Fig. 6J). Taken together, our findings revealed that silencing DEC1 enhances the inhibitory effect of ID4 on GC malignancy in vitro and in vivo.

### ID4 interacts with DEC1 to regulate GC cell proliferation and migration by inhibiting SHH signaling pathway in vitro

**Fig. 7:**
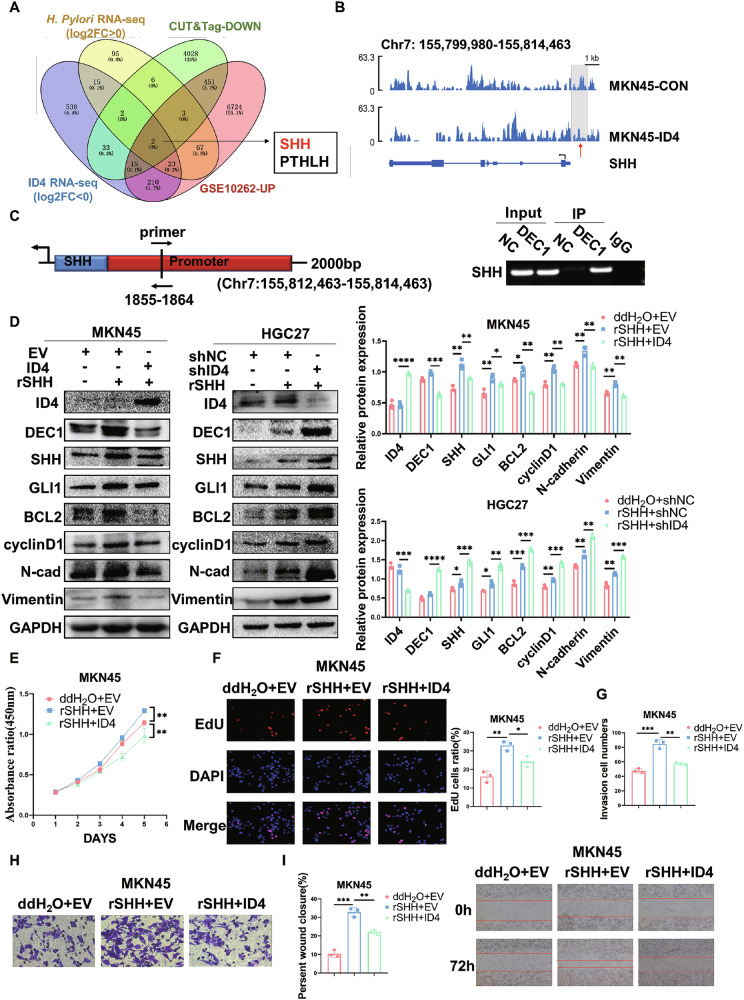
ID4 interacts with DEC1 to regulate GC cell proliferation and migration by inhibiting SHH signaling pathway in vitro. **A** Prediction of ID4-targetd genes through the overlap of ID4 downregulated genes by RNA-seq and CUT&Tag-seq, *H. pylori* infection upregulated genes by RNA-seq and the GEO database (GSE10262). **B** IGV showed the enrichment levels of SHH in MKN45 control and MKN45-ID4 cells in designed genomic regions. **C** ChIP was performed to detect the DEC1 binding region of the SHH promoter. **D** Expression of the indicated SHH signaling pathway molecules (SHH and GLI1), EMT and cell proliferation regulatory proteins (N-cadherin, Vimentin, BCL2 and cyclinD1) with ID4 overexpression/ knockdown when stimulated with 0.5 μg/ml rSHH (measured by using western blot). **E–I** 0.5 μg/ml rSHH was added to MKN45 cells with ID4 overexpression for 6 h. CCK-8, EdU (**E**, **F**) assays detected the proliferative capacity of cells and transwell, wound healing (**G–I**) assays monitored the migration ability of cells. *P < 0.05, **P < 0.01, ***P < 0.001, ****P < 0.0001.

To predict ID4-targeted genes that are associated with *H. pylori* infection, key genes were extracted from ID4 downregulated genes by RNA-seq and CUT&Tag-seq. In addition, we also analyzed upregulated genes in *H. pylori* infection by RNA-seq and a public dataset of mouse gastric epithelial progenitor cells (mGEPs) with *H. pylori* infection (GSE10262). By intersecting the DEGs from the four databases, we finally obtained two genes (SHH and PTHLH) (Fig. 7A). Several studies have suggested that Hedgehog signaling pathway plays a crucial role in the induction and progression of inflammation and neoplastic transformation in *H. pylori* infection [38–40]. To demonstrate the activation status of the SHH pathway, we evaluated the protein expression of SHH, PTCH1, SMO and GLI1 in *H. pylori*-infected mice (n = 6) by Western blot. The results demonstrated that the protein expression levels of SHH, SMO and GLI1 were upregulated in *H. pylori*-infected mice, while that of PTCH1 was downregulated (Fig. S6A). Our findings suggested that *H. pylori* infection induced an increase in SHH levels. The binding of SHH to PTCH1 resulted in the degradation of PTCH1 and the release of PTCH1-mediated inhibition of SMO, leading to SMO activation and subsequently upregulating GLI1 expression. Consequently, our research focused on the SHH gene which plays a pivotal role in the initiation of gastritis in response to *H. pylori* infection. The results of ID4-CUT&Tag sequencing demonstrated that ID4 overexpression impaired SHH transcription at the promoter region (chr7:155812463-155814463) (Fig. 7B). Since ID4 does not directly bind to the promoter region of SHH, we hypothesized that ID4 repressed the transcriptional regulation of SHH by interacting with DEC1. Our analysis of the above SHH promoter region identified a possible DEC1 binding site and designed a pair of primers based on the binding site. The results of the ChIP assay showed that DEC1 could directly bind to the promoter of SHH to influence its expression (Fig. 7C).

To further demonstrate the effect of ID4 on SHH signaling pathway in GC cells, we added human recombinant SHH (rSHH, 0.5 μg/ml) to the cultures of MKN45 and HGC27 cells and incubated for 6 h. Subsequently, western blot analysis revealed that rSHH treatment significantly activated SHH signaling pathway and upregulated the expression of SHH and GLI1 (Fig. S6B). Next, human recombinant SHH was also added to ID4-overexpressed MKN45 cells and ID4-knockdown HGC27 cells to confirm the effect of ID4 on SHH signaling pathway upon stimulation with rSHH. Western blot analysis indicated that ID4 reduced the expression of SHH, GLI1, EMT and cell proliferation regulatory proteins, which were partially restored by treatment with rSHH (Fig. 7D). CCK-8, EdU, transwell and wound healing assays also suggested that ID4 inhibited the proliferation and migration abilities of MKN45 cells, which were promoted upon stimulation with rSHH. Conversely, knockdown of ID4 could further enhance the activation of SHH signaling pathway and promote the proliferation and migration abilities of HGC27 cells (Fig. 7E–I, Fig. S6C–F). Overall, the above results showed that ID4 interacts with DEC1 to inhibit the activation of SHH signaling pathway in vitro.

### ID4 enhances the inhibitory effect of vismodegib on tumor growth and metastasis in vivo

**Fig. 8:**
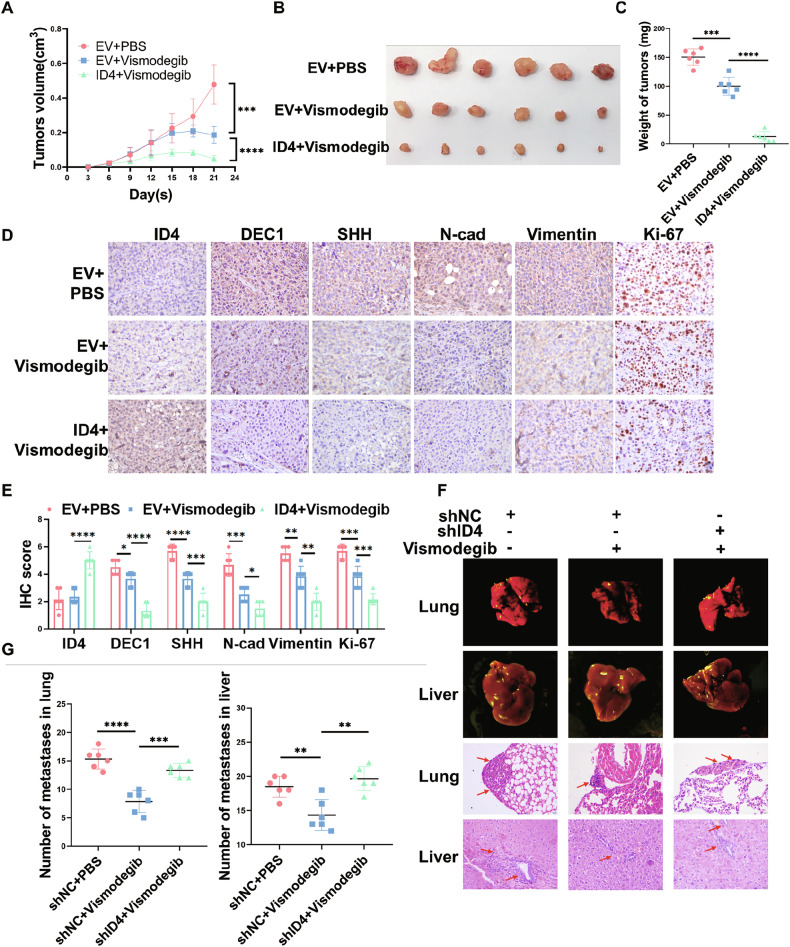
ID4 enhances the inhibitory effect of vismodegib on tumor growth and metastasis in vivo. **A–C** Subcutaneous tumor models (n = 6 mice per group) were established using stable ID4-overexpressed and negative control MKN45 cells treated with vismodegib via gavage (10 mg/kg, once daily, 7 times). Growth curves (**A**) and tumor weights (**C**) were analyzed. Mass images of tumors collected from mice are shown (**B**). IHC staining (**D**) and IHC score (**E**) of protein expression levels of ID4, DEC1, SHH, EMT markers (N-cadherin and vimentin) and Ki-67 in xenograft tumors. **F**, **G** Representative mass images of metastatic tumor nodules (n = 6 mice per group) in the lung and liver of mice and representative H&E images of lung and liver samples from the indicated groups of nude mice (**F**). The number of metastasis tumor nodules in the lung and liver and statistically analyzed (**G**). *P < 0.05, **P < 0.01, ***P < 0.001, ****P < 0.0001.

To investigate whether ID4 inhibits gastric carcinogenesis via SHH signaling pathway in vivo, we performed animal experiments in combination with vismodegib (GDC-0449), which is a potent and orally active inhibitor of SHH signaling pathway that shows significant activities of antitumor. First, we found that ID4 could further enhance the inhibitory effects of vismodegib on the expression of the indicated SHH signaling pathway molecules (SHH and GLI1), EMT and cell proliferation regulatory proteins (N-cadherin, Vimentin, BCL2 and cyclinD1) at the cellular level (Fig. S7A). Subsequently, we subcutaneously injected ID4-overexpressing and negative control MKN45 cells into 6-week-old BALB/c nude mice. These tumor-bearing mice were treated with vismodegib (10 mg/kg) or PBS via gavage for one week. The results showed that treatment with vismodegib inhibited tumor growth, reduced tumor weight and even induced tumor regression, while the combination therapies for ID4 overexpression exhibited more potent effects (*P* < 0.0001, Fig. 8A–C). Next, EMT markers (N-cadherin and vimentin) and Ki-67 IHC staining again showed that overexpression of ID4 could further impair the proliferation and migration of tumor cells when treated with vismodegib (Fig. 8D, E). Additionally, we injected stable ID4-knockdown and negative control HGC27 cells into the tail vein of 5-week-old BALB/c mice, followed by treatment with vismodegib or PBS via gavage once daily for one week. Through collecting their lungs and livers, we observed that knockdown of ID4 could reverse the inhibitory effect of GC metastasis after vismodegib treatment (Fig. 8F, G). Overall, our results revealed that ID4 enhanced the inhibitory effect of vismodegib on GC malignancy in vivo.

## Discussion

The development of GC is the result of a combination of epigenetic, genetic, and environmental factors that result in molecular alterations in cells [41]. *H. pylori* infection is the primary environmental factor that leads to the development of inflammatory disorders and neoplastic transformation of the gastric epithelium [23, 38]. Furthermore, epigenetic abnormalities including DNA methylation, histone modification, and regulation of chromatin structure also play a crucial role in GC development [3]. Gene silencing of tumor suppressor genes mediated by regional methylation in promoter regions has been widely studied in gastric cancer. Numerous studies have highlighted a significant correlation between *H. pylori*-induced aberrant DNA methylation and gastric carcinogenesis [5, 42]. However, mechanisms of *H. pylori*-induced gene silencing of tumor suppressor genes gastric carcinogenesis remain poorly understood.

Although ID4 is known to act as a tumor suppressor in various tumor types, including prostate cancer [43], breast cancer [44], colorectal cancer [45] and lung cancer [46], the ID4 connection in *H. pylori*-induced GC progression is scarcely understood. Here, we found a low expression of ID4 in GC tissues of human and mice and its prognostic value in a public database and our cohort. In addition, we detected a significant decrease in ID4 expression in response to *H. pylori* infection in human and mouse gastric tissues. A few studies have investigated the DNA methylation modulation of ID4 in several tumor tissues [11–13]. We speculate that *H. pylori* infection inhibits ID4 expression by inducing methylation modifications in the ID4 promoter region, which was confirmed by MSP and MethyLight assays. It has been reported that the expression of DNMTs is elevated in various tumor tissues and cell lines, which may be a potential mechanism for the increasing methylation of promoter cytosine and CpG islands of tumor suppressor genes in malignancies [47]. Using the GEPIA online database, we found that only DNMT3B was significantly correlated with ID4 in terms of expression among the three DNMTs. DNMT3B is primarily located in the nucleus and its main function is de novo methylation, which can lead to the inactivation of tumor suppressor genes [48]. Our data confirmed that DNMT3B could bind to the CpG island region in the ID4 promoter and regulate its expression and methylation levels. DNA demethylating agents are now used as a therapeutic approach for hematological tumors and are being developed for solid tumors [49]. However, the two DNA demethylating agents currently in clinical use, DAC and AZA, have dose-limiting toxicities and make it difficult to achieve stable pharmacokinetics. Additionally, their treatment efficacy is also frequently limited by the chemoresistance phenotype of cancer cells [50]. Therefore, novel DNA demethylating agents are currently being sought for use in cancer therapy. Cytotoxin-associated gene A (CagA) is considered one of the bacterial factors of *H. pylori* associated with gastric carcinogenesis. The *H. pylori cag* pathogenicity island encodes a bacterial type IV secretion system and transfers CagA and peptidoglycan peptides into host epithelial cells to activate the transcription factors [51, 52]. Yoshito et al. found that CagA enhanced the expression of DNMT3B in vitro and in vivo [53]. Therefore, we employed an animal model to validate the effect of CagA combined with the DNMT3B-selective inhibitor Nanaomycin A treatment on tumor growth of GC cells. Our research has demonstrated that Nanaomycin A can be an effective therapeutic agent for tumors in vitro and in vivo by demethylating ID4, which provides an effective therapeutic strategy for GC. However, the exact mechanism by which *H. pylori* infection regulates DNMT3B expression has not been investigated. Using the Signaling Pathways Project analysis, we revealed that KLF5 is the transcription factor predicted to bind to the promoter region of DNMT3B in the human stomach. Jennifer et al. have shown that *H. pylori* infection promotes the expression of KLF5 in vitro and in vivo [32]. Based on our results, we speculate that KLF5 regulates DNMT3B expression in response to *H. pylori* infection in GC cell lines. By investigating the upstream regulatory mechanisms of ID4, we demonstrated that its expression was related to the methylation induced by *H. pylori* in a DNMT3B-dependent manner. The present study provides a novel mechanism of ID4 methylation in GC.

Previous studies have indicated that inhibition of DNA binding is the crucial biochemical attribute of ID proteins based on their extensive sequence homology in the HLH motif, which mediates the formation of heterodimers with bHLH transcription factors, impairing DNA binding and bHLH-directed transcription [54]. Numerous studies have demonstrated the interaction of ID proteins with bHLH TFs. Katrin et al. have shown that ID1 and ID3 can interact with TWIST1 (BHLHA38) to regulate fibroblast activation and tissue fibrosis [55]. Furthermore, it has also been proposed that ID2 and ID4 can interact with OLIG1 (BHLHE21) and OLIG2 (BHLHE19) to mediate the inhibitory effects of BMP4 on oligodendroglial differentiation [56]. In this study, RNA sequencing was performed to identify the potential ID4-interacting bHLH TFs in GC. BHLHE40 (DEC1) is the only one among the three TFs that interacts with ID4 and correlates with poor prognosis in GC. Previous and our studies have reported a key role of DEC1 in *H. pylori*-positive GC [57]. DEC1 is a crucial regulator of cellular responses in microenvironment and is involved in cell differentiation, proliferation and apoptosis [58]. Our data suggested that ID4 interacted with DEC1 to co-regulate downstream signaling pathways and silencing DEC1 could further enhance the inhibitory effects of ID4 in GC.

The Hedgehog signaling pathway regulates normal cell growth and differentiation. Its canonical activation occurs through binding of Hh ligands to the transmembrane receptor Patched 1 (PTCH1), which derepresses the receptor Smoothened (SMO) and releases GLI1 to activate target genes [59]. Of the three Hh ligands, SHH is the most potent and broadly expressed HH protein [60]. Numerous studies have reported that *H. pylori* infection in gastric tissue is closely linked with dysregulation of SHH signaling pathway. They suggested that *H. pylori* induced NF-κB activity in a CagA-dependent manner to activate SHH expression [40, 61–63]. Furthermore, SHH regulates growth and differentiation within gastric mucosa and is implicated in the restitution of stem/progenitor cells in damaged gastric mucosa during *H. pylori* infection [64]. Therefore, the role of SHH pathway in the function of ID4 infected by *H. pylori* deserves further investigation. Through RNA-seq and CUT&Tag- seq, we found that SHH is the only gene in the Hh signaling pathway associated with both *H. pylori* infection and ID4. The ChIP assay also showed that DEC1 could directly bind to the promoter of SHH to influence its expression. Therefore, our results showed that ID4 interacts with DEC1 to inhibit the activation of SHH signaling pathway. We performed animal experiments with an orally active inhibitor of SHH signaling pathway, vismodegib, which is an FDA-approved drug for the treatment of basal cell carcinoma. Studies have confirmed that vismodegib could interact with USP28 and USP25 and downregulate the levels of the two enzymes’ substrate proteins c-Myc, Notch1, and Tankyrase-1/2 to inhibit malignant processes in colorectal cancer [65]. Zhao et al. proposed that vismodegib could inhibit the growth of tumors and represent a therapeutic avenue for esophageal squamous cell carcinoma (ESCC) treatment [66]. Additionally, it can also reverse chemotherapy resistance in CD44 ( + ) GC cells [67]. In this study, we found that vismodegib could further enhance the inhibitory effect of ID4 on tumor growth and metastasis in vivo, which provides a novel therapeutic strategy for the treatment of GC.

In the present study, we offered the pioneering evidence that *H. pylori* infection significantly inhibited ID4 expression and increased the methylation level of the ID4 promoter region in GC cell lines and the clinical cohort, which is mediated by KLF5-DNMT3B pathway. Additionally, inhibition of ID4 expression blocked the interaction between ID4 and DEC1, thereby activating the transcriptional activity of DEC1 on SHH to drive gastric cancer progression. In summary, this study provides insights into the mechanisms of *H. pylori*-infected ID4 aberrant methylation and broadens the scope of ID4-mediated transcriptional mechanisms underlying gastric carcinogenesis. Our findings provide a strategic framework for integrating *H. pylori* infection, DNA methylation, and tumor progression, facilitating the optimization of diagnostic accuracy and shedding light on the development of novel therapeutic strategies for the treatment of GC.

## Methods and materials

### Human tissue samples

We collected 95 tumor samples from patients diagnosed with GC in Jinan Central Hospital affiliated with Shandong First Medical University. The *H. pylori* infection in patients was confirmed through a combination of pathological diagnosis, ^13^C urea breath test or serum *H. pylori* antibody test. The study received approval from the Medical Ethics Committee of Jinan Central Hospital affiliated with Shandong First Medical University. Patient samples were collected and processed according to the approved guidelines. Informed consent was obtained from all participating patients. Tissue microarrays (No. ST8019) containing 80 AG tissue samples were purchased from Alina Biotechnology (Xi’an, China).

### Cell culture and reagents

GC cell lines (AGS, MGC803, NCI-N87, MKN28, MKN45 and HGC27) were obtained from the Shanghai Institute of Biochemistry and Cell Biology, Chinese Academy of Science (Shanghai, China). Dulbecco’s Modified Eagle Medium (DMEM) (MACGENE) containing 10% fetal bovine serum (FBS) (Hyclone, USA) was utilized to maintain these cell lines. These cells were then cultured at 37 °C in a humidified 5% CO_2_ incubator (Thermo Fisher Scientific, MA, USA). All cell lines were routinely tested for mycoplasma infection. Short Tandem Repeat (STR) identification was performed on all cell lines.

### Organoid construction

Clinically obtained tumor tissue was trimmed using sterilized scissors and forceps, and the parenchymal portion of the tumor was selected for digestion in primary tumor tissue digestion solution (Mogengel Bio, Xiamen, China). Digestion was terminated through the addition of FBS, and the tissue precipitate was obtained by centrifugation. The precipitate was then treated with erythrocyte lysate (Mogengel Bio) and washed twice with DPBS. The resulting cell precipitates were resuspended in matrix gel (Mogengel Bio) and added to 24-well plates, followed by culture in gastric cancer organoid complete medium (Mogengel Bio).

### Analysis of public databases

The mRNA expression of 408 GC and 211 normal control samples was obtained from The Cancer Genome Atlas (TCGA) database to analyze the ID4 expression using GEPIA. Download the corresponding single-cell data in .h5 format and annotation results from TISCH. Use the R software MAESTRO and Seurat to process and analyze the single-cell data. Graph-based clustering, *t*-distributed stochastic neighbor embedding (t-SNE), was employed. Two GC databases with unpaired adjacent noncancer tissues (GSE54129, GSE66229) were downloaded from the Gene Expression Omnibus (GEO) website. The Kaplan–Meier plotter database was used to verify the correlation between ID4 expression and the prognosis of patients with GC. The correlation analysis of ID4 methylation and mRNA expression was conducted using the online tool cBioPortal. ROC curve analysis was employed to assess the clinical diagnostic efficacy of ID4 methylation in stage and prognosis from TCGA database, *H. pylori* infection status from GSE99553 database and N/T tissues from GSE30601 database.

### *H. pylori* cultures and *H. pylori*-infected cell

*H. pylori* strains 11637, 26695 and SS1 were cultured in Helicobacter Pylori Medium (Hopebio, Qingdao, China) supplemented with 7% defibrinated sheep blood (Hopebio) and *H. pylori* selective supplement (Hopebio) following the manufacturer’s instructions under microaerobic conditions (5% O_2_, 10% CO_2_ and 85% N_2_) at 35 °C. *H. pylori* strains 11637, 26695 and SS1 were used for in vitro co-culture with GC cells at a multiplicity of infection (MOI) of 10, 50 and 100 for 8 h.

### Cell transfection and lentivirus infection

The DNMT3B and KLF5 small interfering RNA (siRNA) or respective negative control (NC) siRNA were purchased from Obio Technology (Shanghai, China). The siRNAs were transfected into cells utilizing Opti-MEM and Lipofectamine 2000 transfection reagent (Invitrogen, CA, USA) at a concentration of 100 nM following the manufacturer’s guidelines. The ID4 and DEC1-silencing lentivirus (shID4, shDEC1), negative control lentivirus (shNC), ID4 and DEC1-overexpressing lentivirus (ID4, DEC1) and empty vector (EV) were constructed by GENECHEM (Shanghai, China). The corresponding sequence information is listed in Table S2.

### Cell transfection of organoids

In the 24-well plate where organoids were to be transfected, matrix gel was added and left to solidify, creating a pre-laid base for subsequent transfection. The ID4 small interfering RNA (siRNA) or respective negative control (NC) siRNA were purchased from Obio Technology (Shanghai, China). The siRNAs were transfected into organoids utilizing gastric cancer organoid complete medium (adding 10% FBS) and Lipofectamine 2000 transfection reagent at a concentration of 100 nM.

### RNA isolation and quantitative real-time polymerase chain reaction (RT-qPCR)

Total RNA was extracted using TRIzol Reagents and reverse-transcribed to cDNA using a HiFiScript gDNA removal cDNA synthesis kit (CWBIO, Taizhou, China). RT-qPCR was performed using the UltraSYBR Mixture (Low ROX; CWBIO) on a LightCycler 480 Real-Time PCR System (Roche, Basel, Switzerland). The sequences of the primers are listed in Table S3. The relative gene expression of mRNAs was calculated using the 2^-ΔΔCt^ method. ACTB was used as the endogenous control to normalize the data.

### Western blotting

Total proteins were extracted using RIPA lysis buffer (Beyotime, Beijing, China) containing phosphatase and protease inhibitors at 4 °C. Subsequently, equivalent amounts of protein were separated by 10% SDS-PAGE and electrophoretically transferred onto a PVDF membrane (Millipore, MA, USA) for 2 h. The blotted membranes were then blocked with 5% non-fat milk for 2 h at room temperature and incubated with the primary antibody overnight at 4 °C. The details of primary antibodies and the respective dilutions are listed in Table S4. After incubation with a horseradish-peroxidase-conjugated secondary antibody, an ECL detection reagent was applied to visualize the binding signals.

### Immunohistochemistry (IHC)

The paraffin-embedded tissues were sectioned, dewaxed and subjected to high- temperature antigen retrieval in EDTA antigenic-retrieval buffer for 15 min. IHC assays were performed using a biotin assay (ZSGB-BIO, Beijing, China) according to the manufacturer’s instructions. The slides were incubated with the primary antibodies, including ID4 (1:200, ABclonal); DEC1, SHH, N-cadherin (1:100, ABclonal); Vimentin (1:500, Proteintech Group); Ki-67 (1:100, ZSGB-BIO) overnight at 4 °C. Next, the sections were incubated with the corresponding secondary antibody and analyzed using a DAB staining kit (Solarbio, Beijing, China). The nucleus was counterstained with hematoxylin and representative areas of each stained tissue section were imaged. The intensity of positive staining was scored on a scale of 0 (negative), 1 (weak), 2 (moderate) and 3 (strong). The proportion of positively stained cells was scored as 1 (<25%), 2 (25–50%), 3 (50–75%) and 4 (>75%). The IHC score was derived by summing up the above two scores.

### Immunofluorescence (IF)

Cells grown on cell slides were subjected to fixation using 4% paraformaldehyde, permeabilizion with 0.5% Triton X-100 (Solarbio), and subsequent blocking with 3% BSA. Primary antibodies ID4 (1:100, ABclonal) and DEC1 (1:100, Santa) were applied to cells, followed by Alexa Fluor-488 and Alexa Fluor-596 fluorescent secondary antibodies (1:400, Thermo Fisher Scientific, USA). Nuclei were counterstained with DAPI. Utilizing a confocal laser scanning microscope (LeicaSP8, Germany), images were captured.

### In vitro anti-GC activity assay

#### Cell viability assessment

Cell proliferation was assayed using the Cell Counting Kit-8 (CCK-8; Dojindo, Japan). MKN45 (4 × 10^3^ cells per well) and HGC27 (3 × 10^3^ cells per well) cell lines were seeded in 96-well plates. Subsequently, we added 100 μl of fresh medium containing 10 μl of CCK-8 solution to cells and incubated the plate for 1 h at 37 °C in a 5% CO_2_ environment. The absorbance at a wavelength of 450 nm was measured using a microplate reader (Bio-Rad, USA).

### EdU staining

The percentage of DNA-replicating cells representing the proliferative state of cells, was determined using the Cell-Light EdU Apollo567 In Vitro Kit (RiboBio, Guangzhou, China) according to the manufacturer’s instructions. The cells were counterstained with DAPI and imaged using a fluorescence microscope (Olympus, Japan).

### Transwell assay

The cell lines MKN45 (2 × 10^5^ cells per well) and HGC27 (1 × 10^5^ cells per well) were suspended in 200 μl of serum-free medium and seeded into a 24-well Boyden chamber (8 μl pore size; Corning, NY, USA) to detect cell migration ability. The lower chamber was supplemented with medium containing 20% FBS. After 24 h (HGC27) or 72 h (MKN45), the cells attached to the bottom of the chambers were fixed with methanol, stained with 0.1% crystal violet and counted under the microscope.

### Wound healing assay

The cell lines MKN45 (2 × 10^5^ cells per well) and HGC27 (1 × 10^5^ cells per well) were seeded into a 24-well plate to form a confluent monolayer, followed by the creation of a uniform scratch with a sterile pipette tip. The phase-contrast images of the scratch were captured at 0 h, 36 h (HGC27) or 72 h (MKN45). Image analysis software was used to measure the wound closure area, and the percentage of closure was calculated in comparison to the initial scratch area.

### Methylation-specific PCR (MSP) and MethyLight Assay

Total DNA was extracted from cells and tissues using Universal Column Genome Extraction Kit (CWBIO). Subsequently, 500 ng of the extracted DNA was subjected to bisulfite conversion using an EZ DNA Methylation-Gold™ kit (Zymo Research, CA, USA) according to the manufacturer’s instructions.

For the MSP assay, 20 ng of the bisulfite-converted DNA was used as a template for PCR using EpiScope® MSP Kit (TaKaRa, Japan). The PCR reactions using the following thermal conditions: 95 °C for 30 s → (98 °C 5 s → 58°C 30 s → 72 °C 40 s) × 45 cycles. The PCR products were separated using 2% agarose gel.

For the MethyLight assay, 10 μl MethyLight reaction mixture contained 5 μl 2X qPCR Probe Master Mix (ABclonal), 10 μM of each primer, 5 μM probe and 20 ng DNA template. The PCR reactions using the following thermal conditions: 95 °C for 5 min → (95 °C 15 s → 55 °C 1 min) × 45 cycles. Purified, methylated human DNA for use as a positive control (Zymo Research, CA, USA), which all cytosines within a CpG dinucleotide context have been enzymatically methylated by M.SssI methyltransferase. Percent of methylated reference (PMR) was calculated according to the formula: PMR = 2^-ΔΔCt^ ×100% (ΔΔCt = ΔCt (ID4-β-actin)_methylated_-ΔCt (ID4-β-actin)_M.SssI_). The primer and probe sequences are shown in Table S5.

### Bisulfite sequencing PCR (BSP)

DNA from cells was isolated and purified utilizing Universal Column Genome Extraction Kit (CWBIO). The methylation state of the ID4 promoter was examined through the application of BSP. It was conducted by Servicebio (Wuhan, China).

### Chromatin immunoprecipitation (ChIP) Assay

ChIP assay was performed using the SimpleChIP® Enzymatic Chromatin IP Kit (Cell Signaling Technology, MA, USA) according to the manufacturer’s protocol. The DNA and proteins were cross-linked and ultrasonically broken into small fragments. The DNA fragments were captured using the anti-DNMT3B and Flag antibodies (Proteintech Group, Wuhan, China) and purified for PCR. The sequences of the primers used for ChIP-qPCR are listed in Table S6. The products were separated via agarose gel electrophoresis.

### Co-immunoprecipitation (Co-IP)

Co-IP assay was conducted using the Pierce^®^ immunoprecipitation kit (Thermo Fisher Scientific, MA, USA) according to the manufacturer’s instructions. EV, ID4 and DEC1 overexpression HEK-293T cell lysates were extracted and incubated with a non-immune IgG for 2 hours at 4 °C to rule out non-specific binding. After that, the lysates were immunoprecipitated with 2 μg of the indicated antibody (Flag and IgG) at 4 °C overnight, followed by the addition of 20 μl A/G PLUS Agarose beads at 4 °C for 4 h. The beads were separated and washed with cold PBS. The coprecipitates were electrophoresed on SDS-PAGE and further analyzed through immunoblotting.

### Cleavage under targets & tagmentation (CUT&Tag) sequencing

According to the manufacturer’s protocol, CUT&Tag was performed using the Hyperactive Universal CUT&Tag Assay Kit for Illumina (Vazyme Biotech, TD903). Flag-tagged ID4 overexpression MKN45 cell lysate was incubated with 10 μl of prewashed ConA Beads and 2 μg of Flag antibody. DNA was fragmented and extracted for amplification. After amplification, all libraries were sequenced using Illumina HiSeq X Ten, and the bigWig tracks were visualized in the Integrative Genomic Viewer genome (IGV) browser.

### Animal studies

#### *H. pylori*-infected mouse models

The *H. pylori* gavage cohort containing 40 4-week-old C57BL/6 mice which were divided into two groups randomly (n = 20/ group) and infected by oral gavage with SS1 strain (1 × 10^9^ colony-forming units/ 200 μl/ mouse) or PBS for 4 months. Finally, all mice were sacrificed and the stomach of mice were dehydrated, fixed and subjected to IHC staining.

### Nanaomycin A treatment assay

100 μl tumor cell suspension containing 7 × 10^6^ MKN45-CagA cells or control cells was subcutaneously inoculated into the left flank of 5-week-old BALB/c nude mice. The mice were then randomly divided into four groups (n = 6/ group). For Nanaomycin A treatment, mouse xenografts were allowed to grow for 1 week prior to intratumoral injection of 1 μM/ 50 μl Nanaomycin A twice a week. Tumor growth was monitored every 2 days. Tumor volume was calculated as volume = (length × width^2^) /2. After 14 days, the mice were sacrificed and tumor tissues were harvested.

### Subcutaneous tumor mouse models

100 μl tumor cell suspension containing 7 × 10^6^ MKN45 cells was subcutaneously inoculated into the left flank of 6-week-old BALB/c nude mice to establish the subcutaneous tumor mouse models. Once tumors from the ID4-overexpression and control groups (n = 12) reached ≈ 0.1 cm^3^, 6 mice were randomly selected for vismodegib (10 mg/kg) or PBS via gavage for one week. Tumor growth was monitored every 3 days. After 21 days, the mice were sacrificed and tumor tissues were harvested for IHC staining.

### Tail vein metastasis mouse models

200 μl tumor cell suspension containing 2 × 10^6^ HGC27 cells was injected into the tail vein of 5-week-old BALB/c nude mice. Similarly, 6 mice from the ID4 knockdown and negative control groups were randomly selected for vismodegib (10 mg/kg) or PBS via gavage for one week. In case of complications such as pulmonary embolism leading to death, these mice were excluded from the study. After 4 weeks, the mice were sacrificed and dissected to observe their lung and liver tumor metastatic nodules. The livers and lungs of the mice were imaged using a stereoscopic microscope (Olympus) and subjected to hematoxylin and eosin (H&E) staining (Solarbio) according to the manufacturer’s instructions.

### Organoid-based Xenograft (PDOX) Models

Well-growing organoids were isolated from the matrix gel, collected by centrifugation, and washed with DPBS. The extracted organoids were injected subcutaneously to establish a subcutaneous tumor model, with the addition of a high-concentration matrix gel during injection to facilitate tumor formation, using a minimum of 1 × 10^6^ cells. We harvested P0 generation tumor tissue and conducted P1 generation tumor transplantation experiments in similar nude mice. Tumor establishment was followed by AAV- ID4 (1.6 × 10^11^ vg/mouse) intratumoral injection two weeks later to assess its impact on tumorigenesis. Administer two injections with an interval of three days between each dose. After 14 days, the mice were sacrificed and tumor tissues were harvested.

### Statistics

All data were presented as mean ± standard deviation and were analyzed using GraphPad Prism 8.0 software. Statistical significance between groups was determined with Student’s *t*-test. Survival curves were calculated by the Kaplan–Meier survival method and compared by the log-rank test. Spearman correlation was used to determine the expression correlation of two genes. A value of *P* < 0.05 was regarded as statistically significant. Experiments were performed independently at least three times. (*P < 0.05, **P < 0.01, ***P < 0.001 and ****P < 0.0001).

## Supplementary information

Supplementary files

Original western blots

## Data Availability

The data that support the findings of this study are available in the supplementary material of this article.
