# Supplementary material for: Helicobacter pylori-induced aberrant methylation of ID4 mediated by DNMT3B drives gastric cancer progression via DEC1-SHH signaling pathway
Source: Cell Death Dis. 2025 Oct 7;16(1):713. doi: 10.1038/s41419-025-08042-9 (PMC12504463; doi:10.1038/s41419-025-08042-9)
Supplement: Supplementary file 1 — Supplementary files [file 41419_2025_8042_MOESM1_ESM.docx]

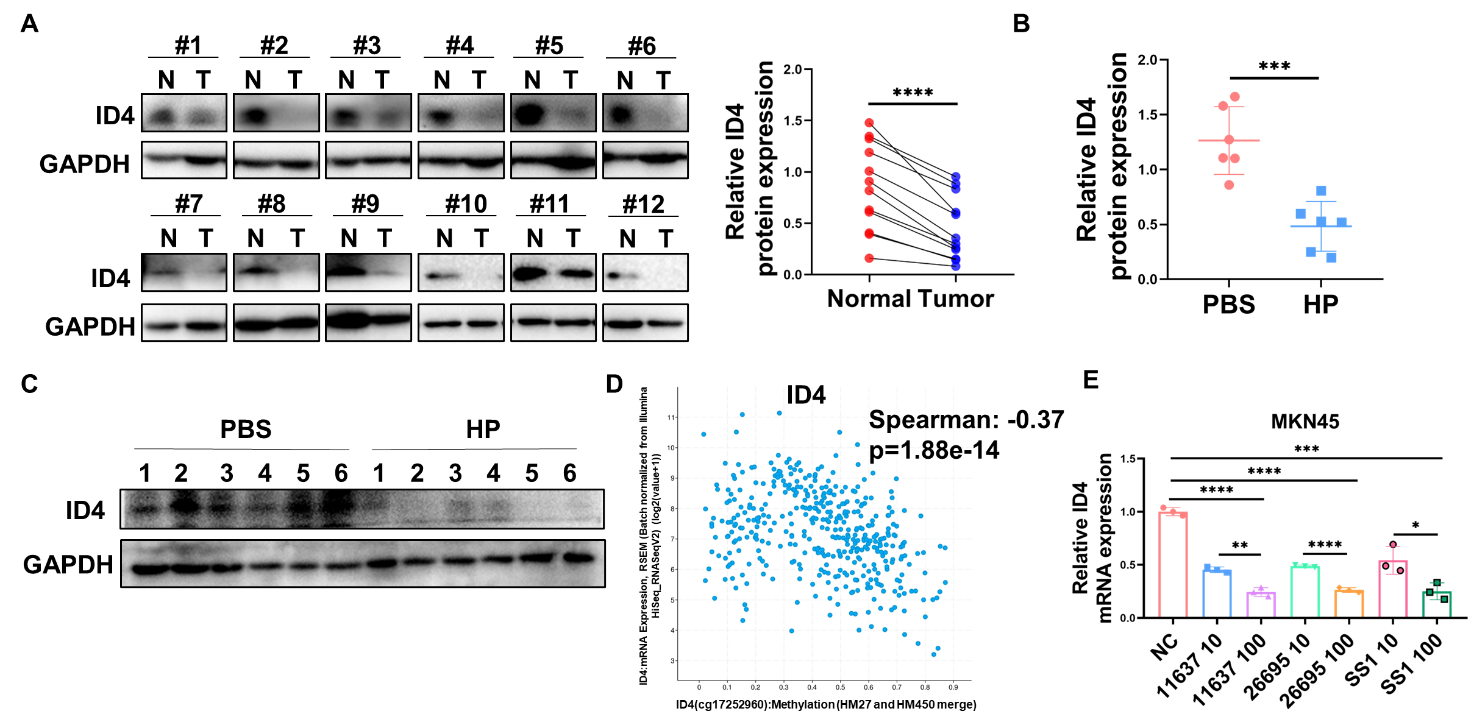


**Figure S1****.** **A Protein expression of ID4 in paired GC tissues and adjacent normal tissues (n=12). B, C** Western blot analysis of ID4 protein expression in the PBS group (n=6) and HP (SS1 strain)-infected (n=6) mice. **D** cBioPortal analysis revealed that ID4 expression is significantly correlated with its DNA methylation level. **E** RT-qPCR detection of ID4 mRNA expression after *H. pylori* infection at an MOI of 10, 50 and 100.

**
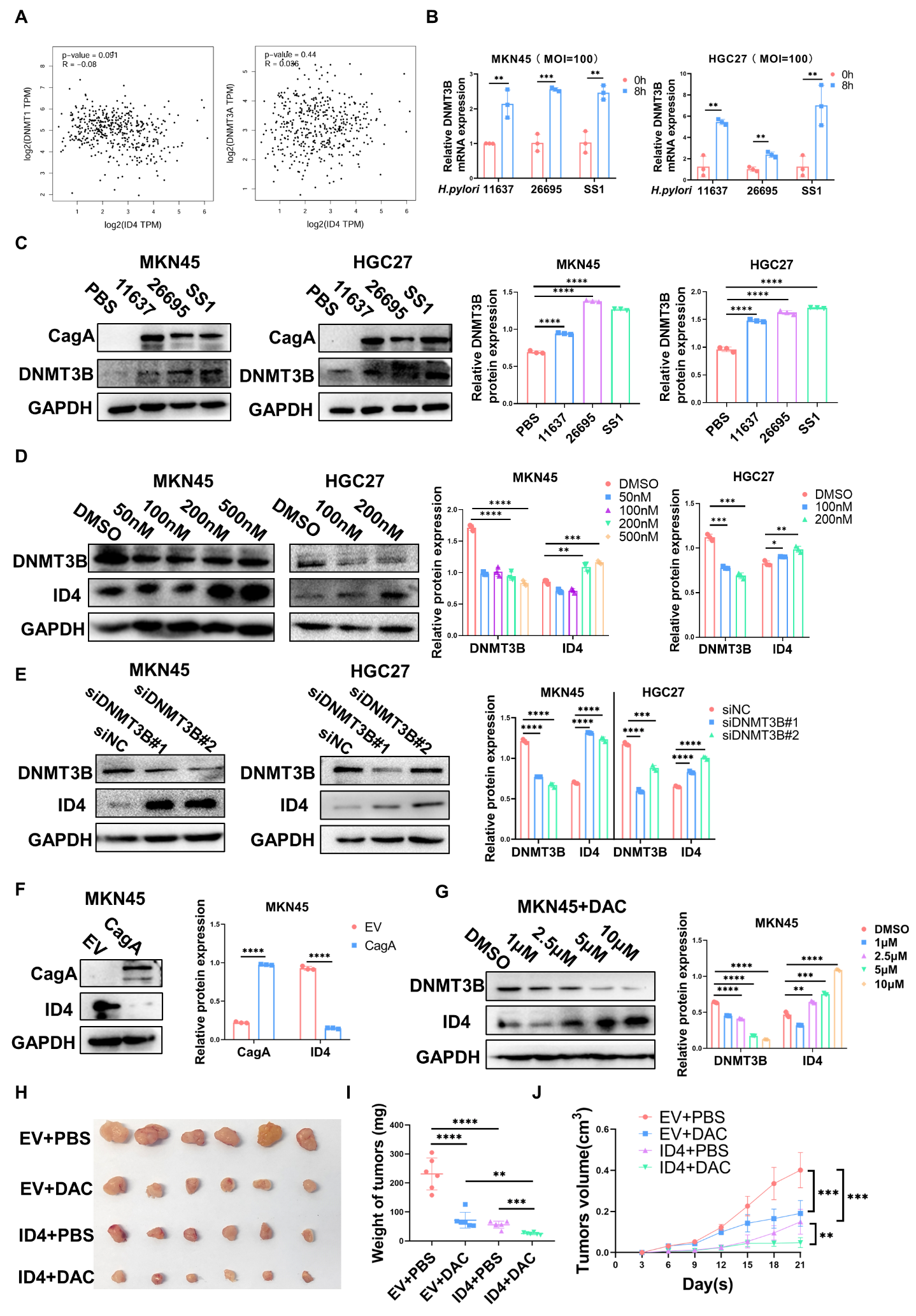
**

**Figure S2.** **A** GEPIA analysis revealed that ID4 expression was not significantly correlated with DNMT1 and DNMT3A in patients with GC. **B** RT-qPCR detection of DNMT3B mRNA expression after infection of the GC cell lines, MKN45 and HGC27 with *H. pylori* 11637, 26695 and SS1 for 8 h (MOI=100). **C** Western blot analysis of DNMT3B protein expression in MKN45 and HGC27 cells infected with *H. pylori* 11637, 26695 and SS1. **D, E** Western blot analysis of ID4 and DNMT3B in MKN45 and HGC27 cells treated with different concentrations of Nanaomycin A (DNMT3B inhibitor) for 24 h or transfected with DNMT3B siRNA (siDNMT3B). **F** Western blot analysis of ID4 protein expression in CagA-overexpression GC cells. **G** Western blot analysis of ID4 and DNMT3B in MKN45 cells treated with different concentrations of DAC. **H** The effects of ID4 combined with DAC treatment on tumor growth of MKN45 cells (n=6 mice per group). **I, J** Tumor weight (I) and tumor growth curve (J) were measured.

**
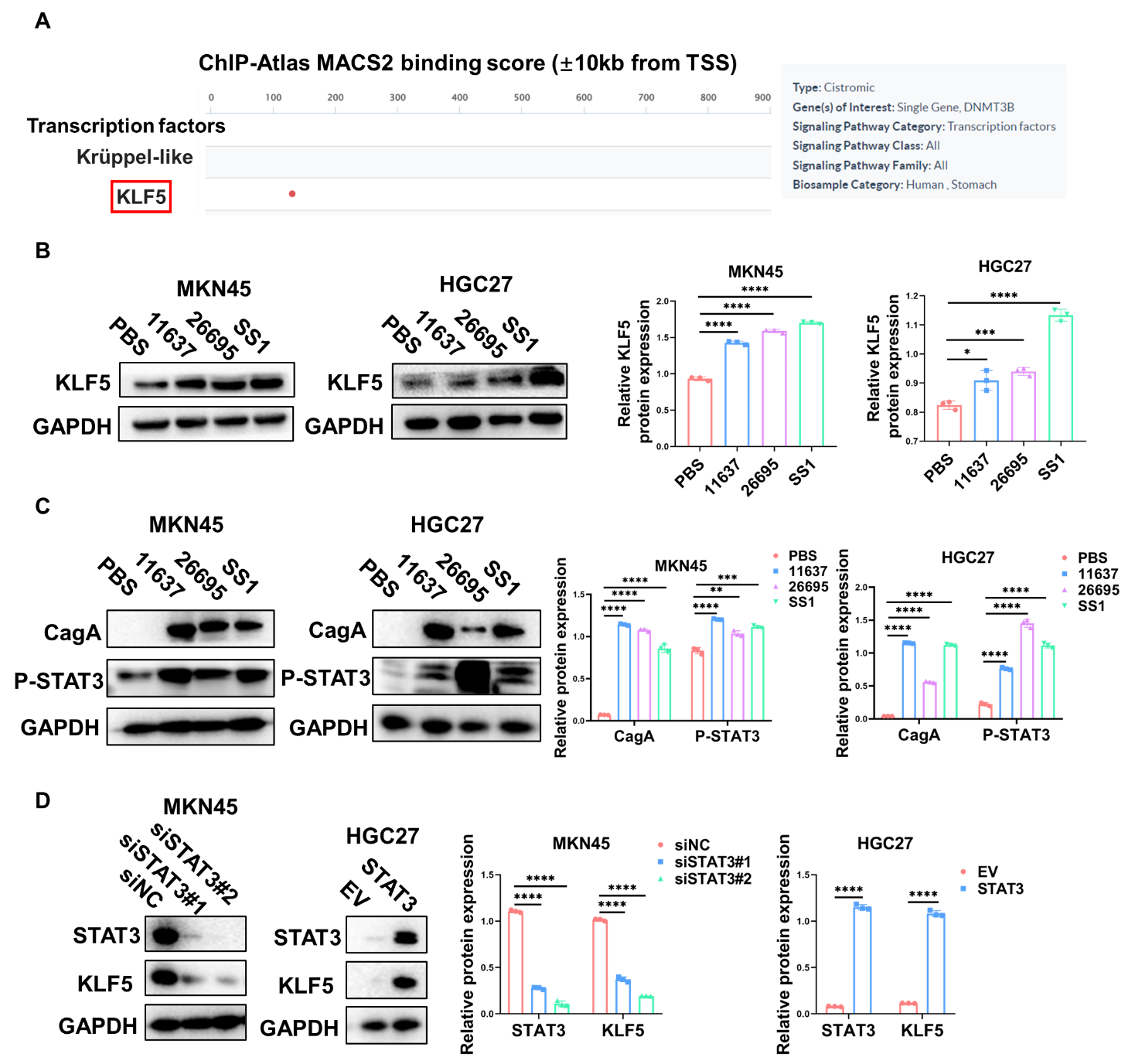
**

**Figure S3.** **A** Prediction of the transcription factor of DNMT3B via the Signaling Pathways Project analysis. **B** Western blot analysis of KLF5 protein expression in MKN45 and HGC27 cells infected with *H. pylori* 11637, 26695 and SS1. **C** Western blot analysis of phosphorylation protein expression level of STAT3 after *H. pylori* infection in MKN45 and HGC27 cells. **D** Western blot analysis of KLF5 expression in STAT3-knockdown and STAT3-overexpression GC cells.

**
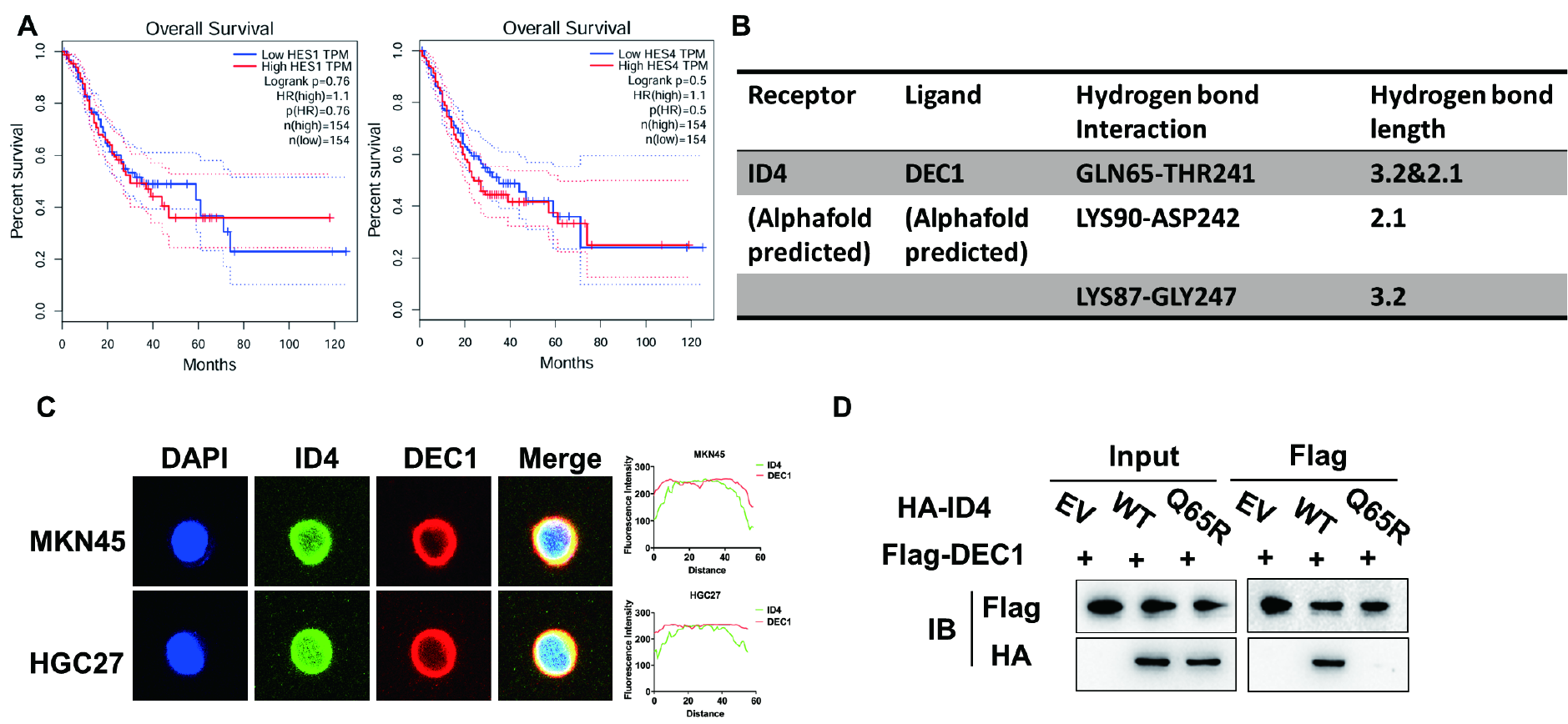
**

**Figure S4.** **A** Overall survival curves corresponding to HES1 and HES4. **B** Alphafold predictive analysis of the binding sites between ID4 and DEC1. **C** The immunofluorescence co-localization analysis of ID4 and DEC1. **D** Co-IP analysis of the impact of ID4 mutant (Q65R) on the binding between ID4 and DEC1.

**
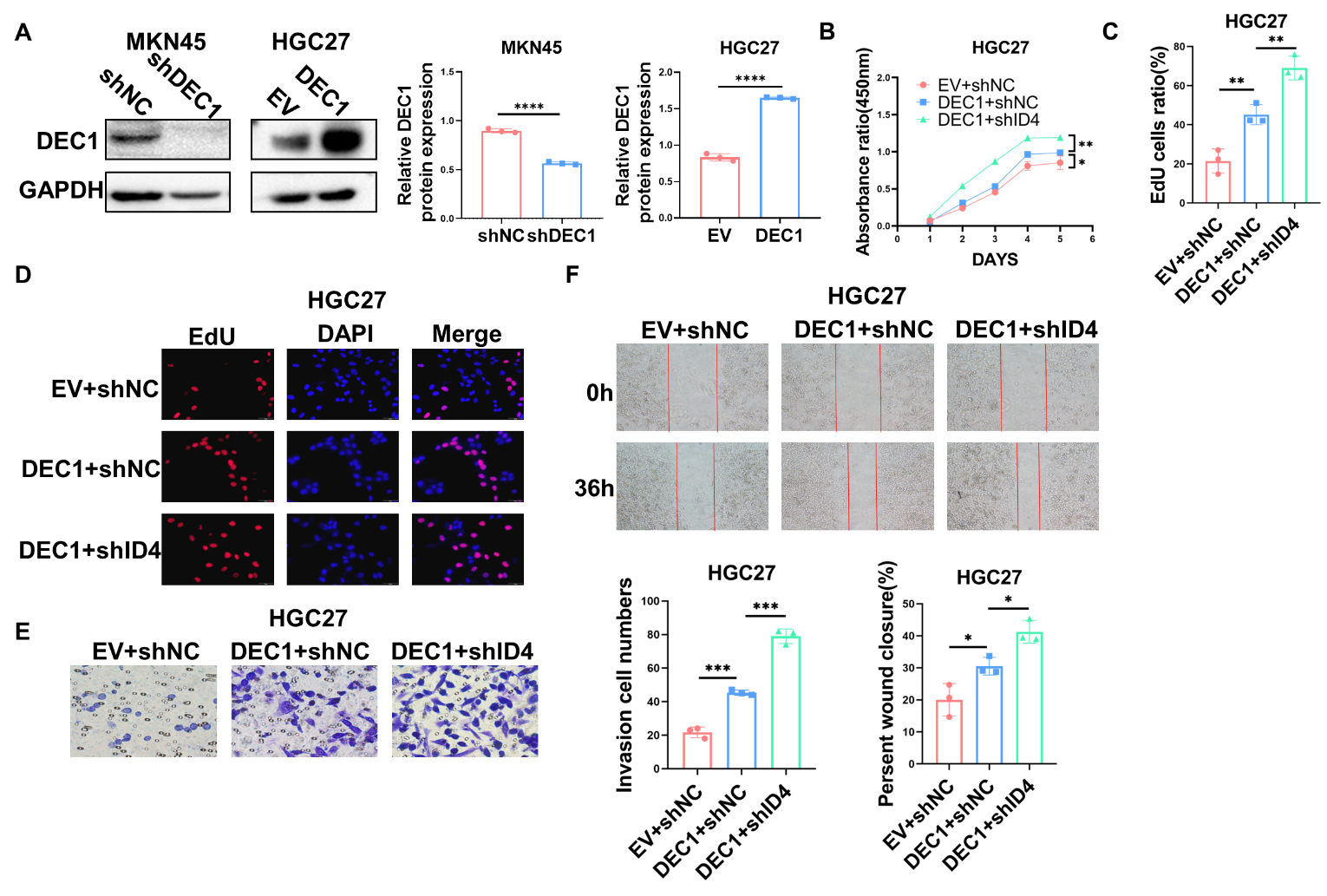
**

**Figure S5. A** Western blot analysis of the transfection efficiency of shRNA-mediated DEC1 repression in MKN45 cells or overexpression of DEC1 in HGC27 cells. **B-D** The effects of ID4 and DEC1 on proliferative ability of GC cells were measured by CCK-8 assays (B) and EdU assays (D). **E, F** The effects of ID4 and DEC1 on migration ability of GC cells were determined by transwell assays (E) and wound healing assays (F).

**
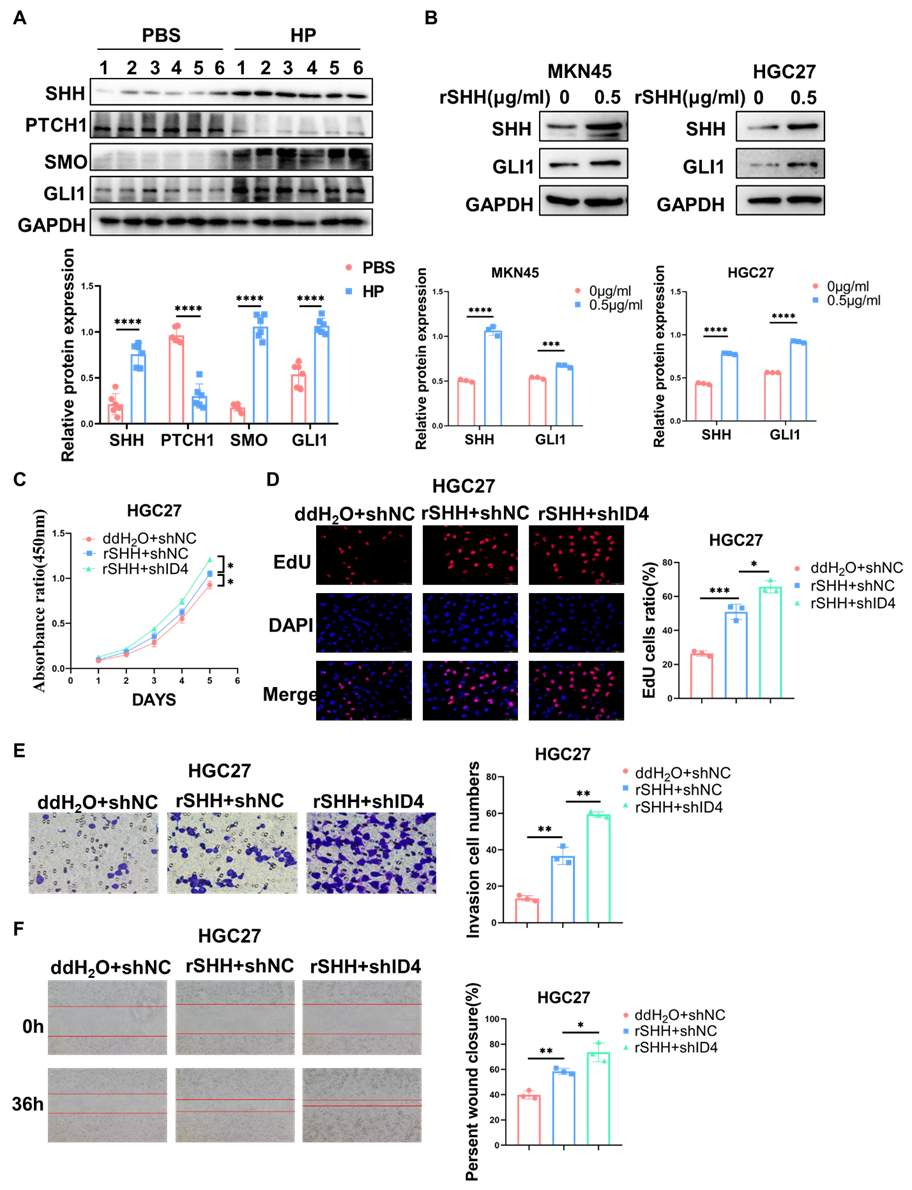
**

**Figure S6.** **A** The expressions of SHH, PTCH1, SMO and GLI1 in *H. pylori*-infected mice (n=6). **B** Western blot analysis of SHH and GLI1 protein expression in MKN45 and HGC27 cells after 0.5 μg ml^-1^ human recombinant SHH (rSHH) stimulation for 6 h. **C-F** 0.5 μg ml^-1^ rSHH was added to HGC27 cells with ID4 knockdown for 6 h. CCK-8, EdU (**C, D**) assays detected the proliferative capacity of cells and transwell, wound healing (**E, F**) assays monitored the migration ability of cells.

**
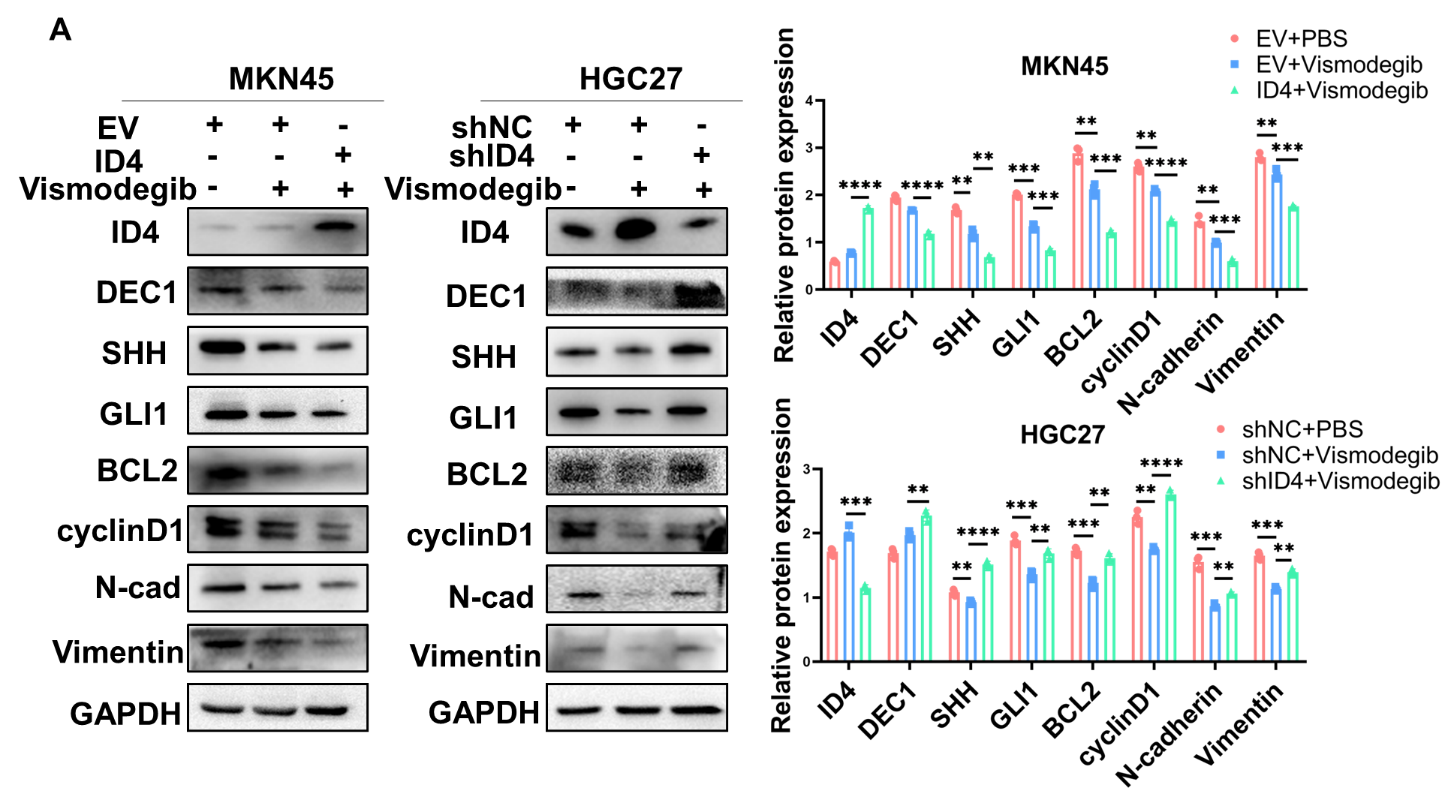
**

**Figure S7.** **A** Expression of DEC1, SHH, GLI1, EMT and cell proliferation regulatory proteins with ID4 overexpression/ knockdown cells treated with 10 μM vismodegib (measured by western blot).

**Table S1.** Correlation between ID4 and clinicopathologic parameters in GC.

| Characteristic |  | Expression of ID4 | |  |  |
| --- | --- | --- | --- | --- | --- |
|  |  | High | Low | χ2-test | *p* value |
| Gender | Male | 46 | 32 | 0.121 | 0.729 |
|  | Female | 7 | 6 |  |  |
| *H. pylori* infection | HP+ | 18 | 24 | 7.095 | 0.008* |
|  | HP- | 32 | 13 |  |  |
| Age | >60 years | 37 | 30 | 0.9513 | 0.329 |
|  | ≤60 years | 16 | 8 |  |  |
| Differentiation | G1 G2 | 21 | 6 | 8.112 | 0.004* |
|  | G3 | 22 | 28 |  |  |
| T classification | T1 T2 | 20 | 6 | 5.009 | 0.025* |
|  | T3 T4 | 29 | 28 |  |  |
| N classification | Positive | 32 | 30 | 4.399 | 0.036* |
|  | Negative | 17 | 5 |  |  |
| M classification | Positive | 2 | 1 | 0.079 | 0.779 |
|  | Negative | 48 | 34 |  |  |
| Clinical staging | ⅠⅡ | 24 | 5 | 10.870 | 0.001* |
|  | Ⅲ Ⅳ | 25 | 30 |  |  |

**Table S2.** Target sequences of siRNA used in this study.

| Gene symbol | Target sequences (5’→3’) |
| --- | --- |
| siKLF5#1 | GAGAATAGGTATGGTCAAA |
| siKLF5#2 | CTGTCTTGATCGAGTTATA |
| siDNMT3B#1 | GGAGGUGUCCAGUCUGCUAAGTT |
| siDNMT3B#2 | CGAGGUCUCUGCAGACAAACUTT |

**Table S3.** PCR primers sequences.

| Name | Primer sequences (5' → 3') |
| --- | --- |
| ID4 (Forward) | TCCCGCCCAACAAGAAAGTC |
| ID4 (Reverse) | CCAGGATGTAGTCGATAACGTG |
| DNMT3B (Forward) | AGGGAAGACTCGATCCTCGTC |
| DNMT3B (Reverse) | GTGTGTAGCTTAGCAGACTGG |
| ACTB (Forward) | TTCCTTCCTGGGCATGGAGTCC |
| ACTB(Reverse) | TGGCGTACAGGTCTTTGCGG |

**Table S4.** Antibodies utilized in Western Blot.

| Antibody | Dilution ratio | Source |
| --- | --- | --- |
| Anti-ID4 | 1:1000 | ABclonal, Wuhan, Hubei, China |
| Anti-GAPDH | 1:5000 | Proteintech Group, Chicago, IL, USA |
| Anti-CagA | 1:800 | GeneTex, San Antonio, TX, USA |
| Anti-DNMT3B | 1:500 | ABclonal, Wuhan, Hubei, China |
| Anti-KLF5 | 1:1000 | ABclonal, Wuhan, Hubei, China |
| Anti-N-cadherin | 1:1000 | ABclonal, Wuhan, Hubei, China |
| Anti-Vimentin | 1:2000 | Proteintech Group, Chicago, IL, USA |
| Anti-BCL2 | 1:2000 | Proteintech Group, Chicago, IL, USA |
| Anti-cyclinD1 | 1:10000 | Proteintech Group, Chicago, IL, USA |
| Anti-BHLHE40(DEC1) | 1:1000 | ABclonal, Wuhan, Hubei, China |
| Anti-SHH | 1:1000 | ABclonal, Wuhan, Hubei, China |
| Anti-GLI1 | 1:5000 | Proteintech Group, Chicago, IL, USA |
| Anti-STAT3 | 1:2000 | Proteintech Group, Chicago, IL, USA |
| Anti-P-STAT3 | 1:1000 | ABclonal, Wuhan, Hubei, China |
| Anti-SMO | 1:1000 | Proteintech Group, Chicago, IL, USA |
| Anti-PTCH1 | 1:1000 | Epizyme, Shanghai, China |

**Table S5.** Primers and probe sequences of MSP and MethyLight.

| Experimental name | Gene name | Primer sequences (5' → 3') |
| --- | --- | --- |
| MSP | ID4-M1 (Forward) | ATTTTTCGTTTATTTTTTTATTCGG |
| MSP | ID4-M1 (Reverse) | CGCAACTATATTTATAAAACCGTACG |
| MSP | ID4-U1 (Forward) | ATTTTTTGTTTATTTTTTTATTTGG |
| MSP | ID4-U1 (Reverse) | CACAACTATATTTATAAAACCATACACC |
| MSP | ID4-M2 (Forward) | TGTTATTAGGAATGGTAGGGTATTC |
| MSP | ID4-M2 (Reverse) | ACGAAAAACTACGAAAATATACGAC |
| MSP | ID4-U2 (Forward) | GTGTTATTAGGAATGGTAGGGTATTTG |
| MSP | ID4-U2 (Reverse) | AATTCCTACAAAAAACTACAAAAATATACA |
| MethyLight | ID4-M(Forward) | ATTTTTCGTTTATTTTTTTATTCGG |
| MethyLight | ID4-M (Reverse) | CGCAACTATATTTATAAAACCGTACG |
| MethyLight | ID4-M (Probe) | ACGCCCCGAAAACGAAACCAACG |
| MethyLight | ACTB (Forward) | TGGTGATGGAGGAGGTTTAGTAAGT |
| MethyLight | ACTB (Reverse) | AACCAATAAAACCTACTCCTCCCTTAAA |
| MethyLight | ACTB (Probe) | ACCACCACCCAACACACAATAACAAACACA |

MSP：Methylation-specific PCR

**Table S6.** ChIP-qPCR primers sequences.

| Name | Primer sequences (5' → 3') |
| --- | --- |
| DNMT3B-ID4 site1 (Forward) | CTTGAACAGCGCGTCTTTCTT |
| DNMT3B-ID4 site1 (Reverse) | GAGAGCGAAGTTCCTGCGAA |
| KLF5-DNMT3B site1 (Forward) | CTCCCTCAGTCAGTCCATGC |
| KLF5-DNMT3B site1 (Reverse) | GCCTGTCATCCTGCTTTGGA |
| DEC1-SHH site1 (Forward) | CTCCCGCCCACCTTTATCTT |
| DEC1-SHH site1 (Reverse) | TATTATAGCTGCCAGGGGCG |
