# Supplementary material for: Helicobacter pylori-induced aberrant methylation of ID4 mediated by DNMT3B drives gastric cancer progression via DEC1-SHH signaling pathway
Source: Cell Death Dis. 2025 Oct 7;16(1):713. doi: 10.1038/s41419-025-08042-9 (PMC12504463; doi:10.1038/s41419-025-08042-9)
Supplement: Supplementary file 2 — Original western blots [file 41419_2025_8042_MOESM2_ESM.pptx]

## Slide 1
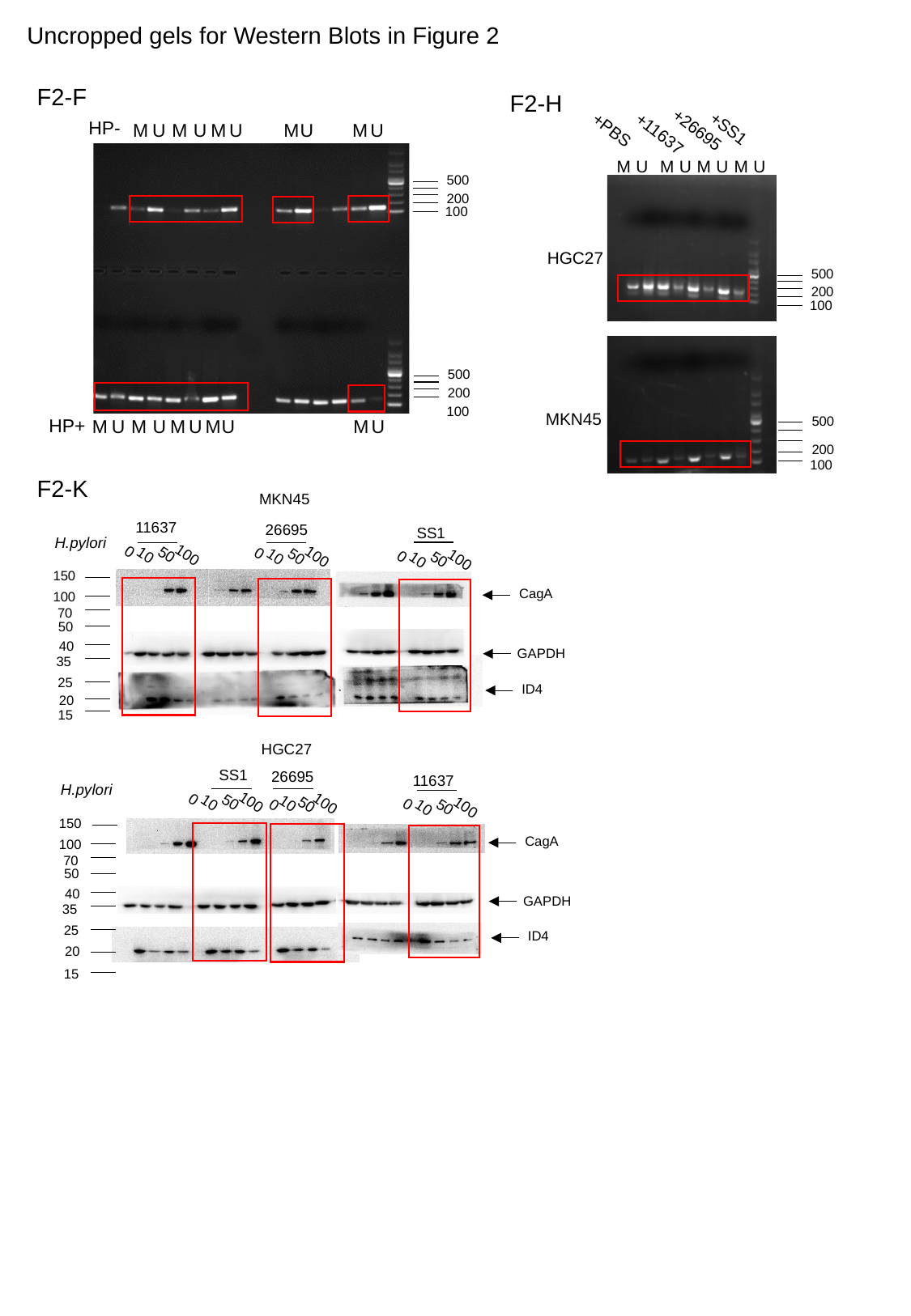

Uncropped gels for Western Blots in Figure 2
F2-F
F2-H
HP-
M
U
M
U
M
U
M
U
M
U
+26695
+SS1
+PBS
+11637
M
U
M
U
M
U
M
U
500
200
100
HGC27
500
200
100
500
200
100
MKN45
500
HP+
M
U
M
U
M
U
M
U
M
U
200
100
F2-K
MKN45
11637
26695
SS1
H.pylori
50
100
0
10
50
100
0
10
50
100
0
10
150
CagA
100
70
50
40
GAPDH
35
25
ID4
20
15
HGC27
SS1
26695
11637
H.pylori
50
100
0
10
10
100
50
50
100
0
10
0
150
CagA
100
70
50
40
GAPDH
35
25
ID4
20
15

## Slide 2
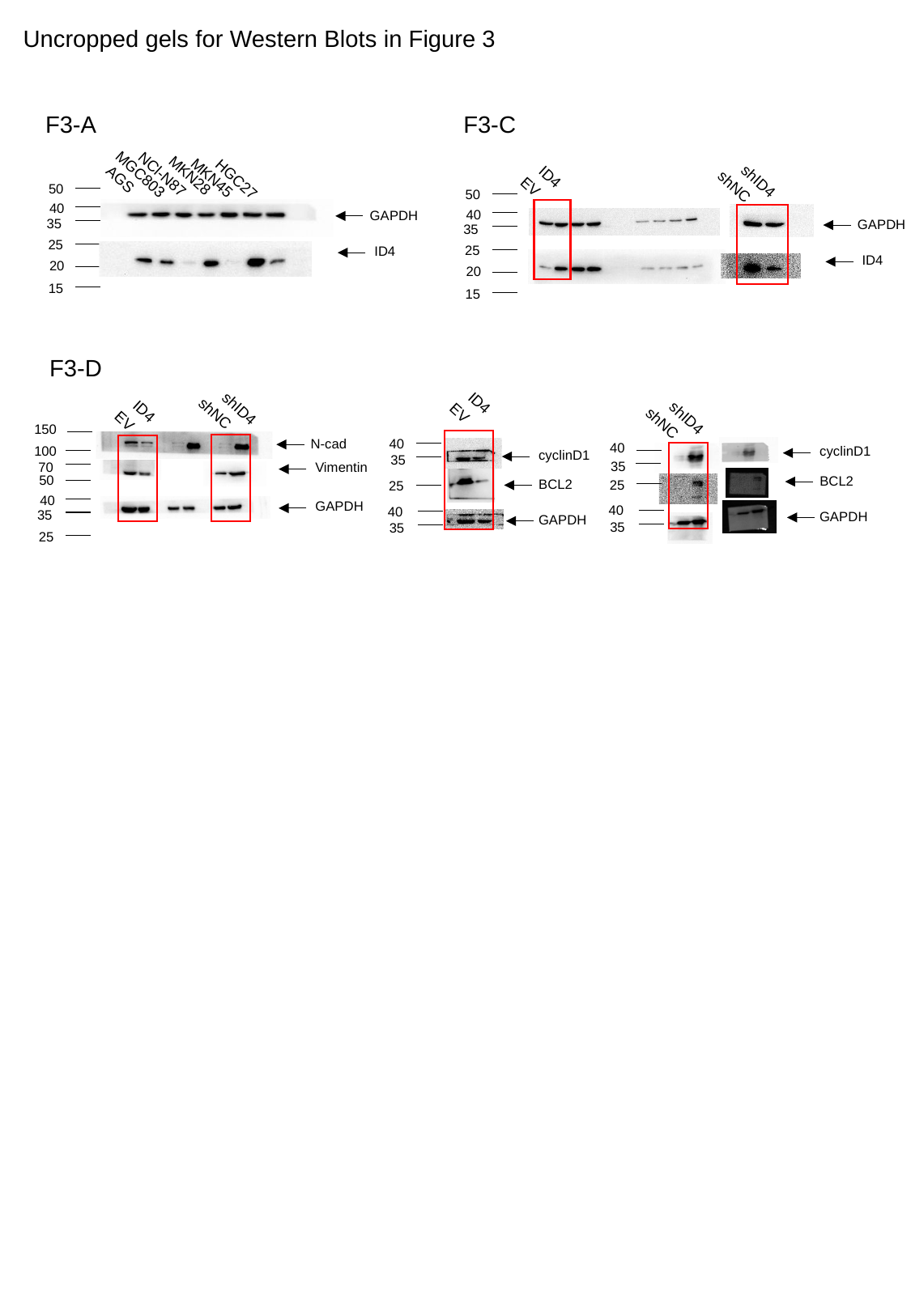

Uncropped gels for Western Blots in Figure 3
F3-A
F3-C
MGC803
NCl-N87
MKN28
ID4
HGC27
MKN45
shID4
AGS
shNC
EV
50
50
40
40
GAPDH
35
GAPDH
35
25
25
ID4
ID4
20
20
15
15
F3-D
ID4
shID4
EV
ID4
shNC
shID4
EV
shNC
150
40
N-cad
40
100
cyclinD1
cyclinD1
35
35
70
Vimentin
50
BCL2
BCL2
25
25
40
GAPDH
40
40
35
GAPDH
GAPDH
35
35
25

## Slide 3
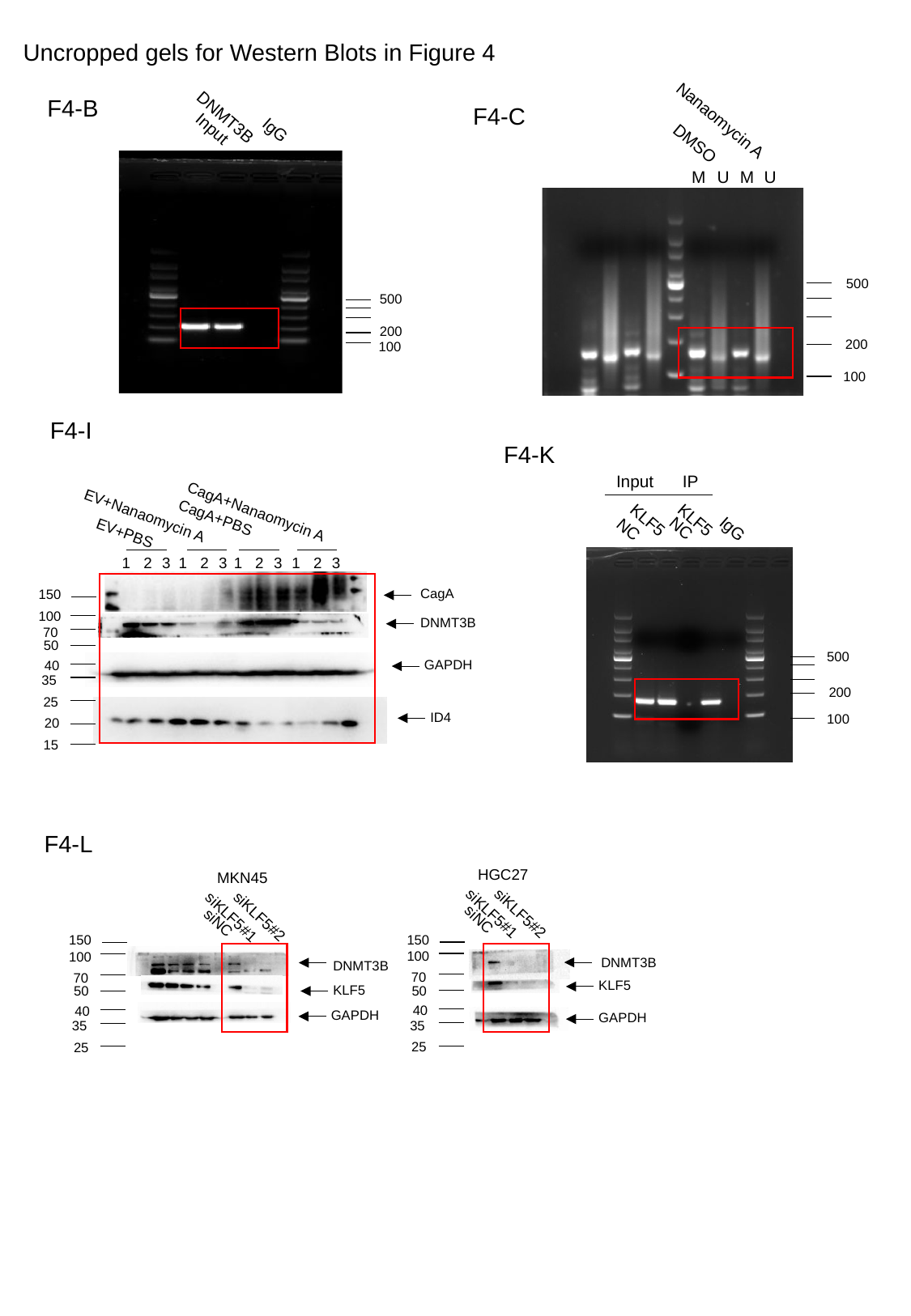

Uncropped gels for Western Blots in Figure 4
F4-B
F4-C
DNMT3B
Nanaomycin A
Input
IgG
DMSO
M
U
M
U
500
500
200
200
100
100
F4-I
F4-K
Input
IP
CagA+Nanaomycin A
EV+Nanaomycin A
CagA+PBS
KLF5
KLF5
 NC
 NC
IgG
EV+PBS
1
2
3
1
2
3
1
2
3
1
2
3
CagA
150
100
DNMT3B
70
50
500
GAPDH
40
35
200
25
ID4
100
20
15
F4-L
HGC27
MKN45
siKLF5#1
siKLF5#2
siKLF5#1
siKLF5#2
siNC
siNC
150
150
100
100
DNMT3B
DNMT3B
70
70
KLF5
KLF5
50
50
40
40
GAPDH
GAPDH
35
35
25
25

## Slide 4
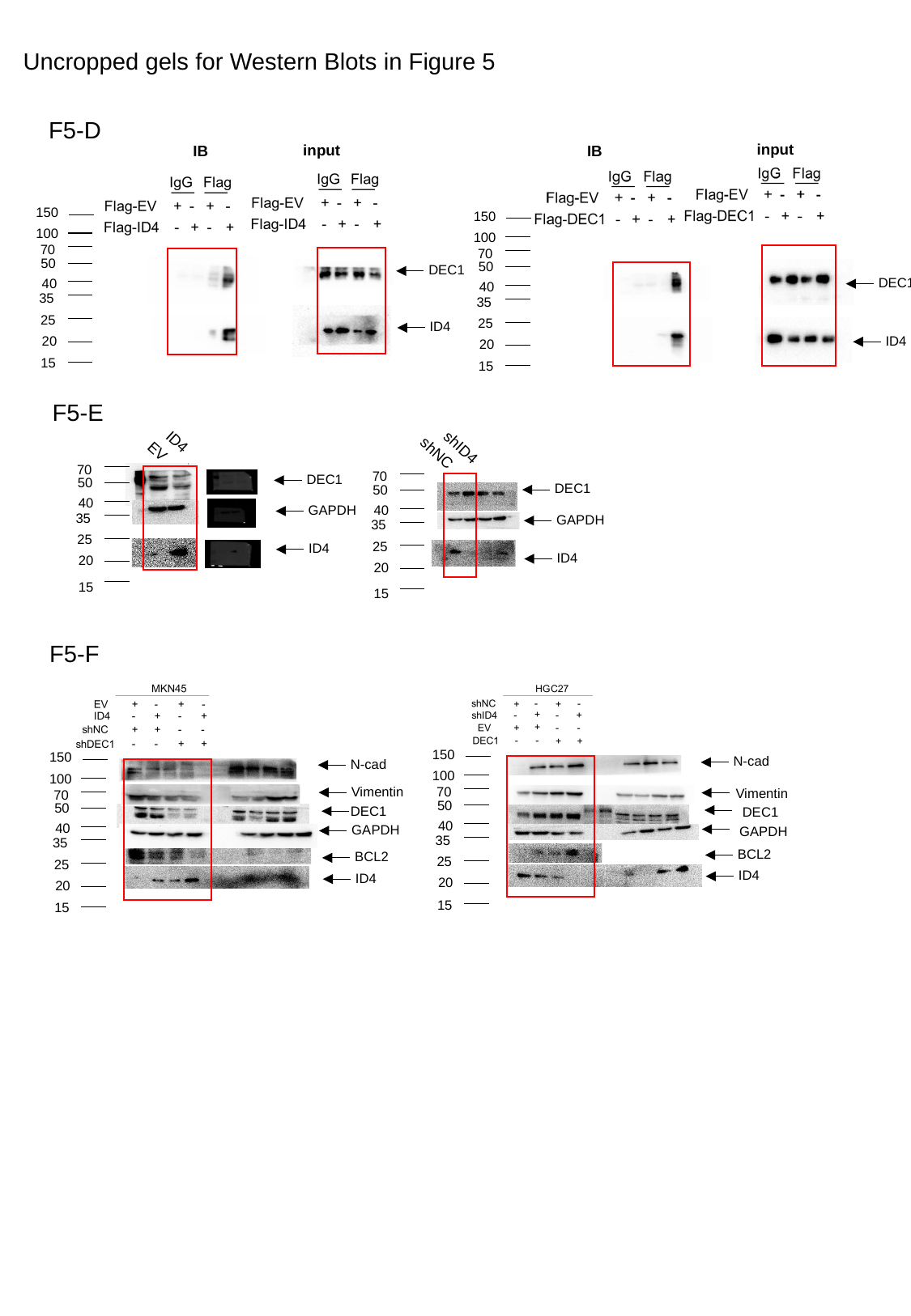

Uncropped gels for Western Blots in Figure 5
F5-D
input
input
IB
IB
150
150
100
100
70
70
50
50
DEC1
DEC1
40
40
35
35
25
25
ID4
20
ID4
20
15
15
F5-E
ID4
shID4
EV
shNC
70
70
DEC1
50
DEC1
50
40
GAPDH
40
35
GAPDH
35
25
25
ID4
ID4
20
20
15
15
F5-F
150
150
N-cad
N-cad
100
100
Vimentin
70
Vimentin
70
50
50
DEC1
DEC1
40
40
GAPDH
GAPDH
35
35
BCL2
BCL2
25
25
ID4
ID4
20
20
15
15

## Slide 5
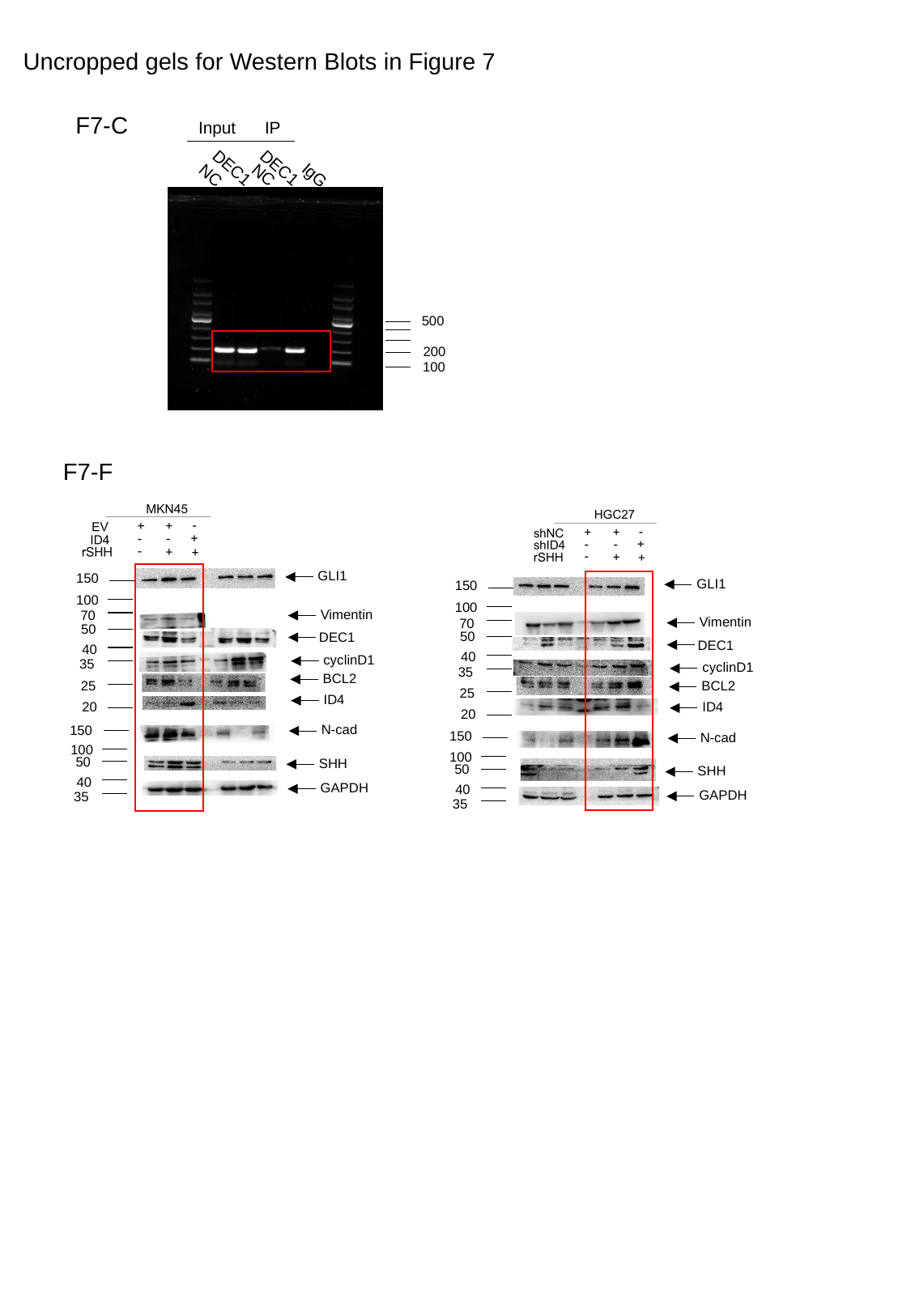

Uncropped gels for Western Blots in Figure 7
F7-C
Input
IP
DEC1
DEC1
 NC
 NC
IgG
500
200
100
F7-F
GLI1
150
GLI1
150
100
100
Vimentin
70
Vimentin
70
50
50
DEC1
DEC1
40
40
cyclinD1
35
cyclinD1
35
BCL2
25
BCL2
25
ID4
20
ID4
20
N-cad
150
150
N-cad
100
100
50
SHH
50
SHH
40
GAPDH
40
GAPDH
35
35

## Slide 6
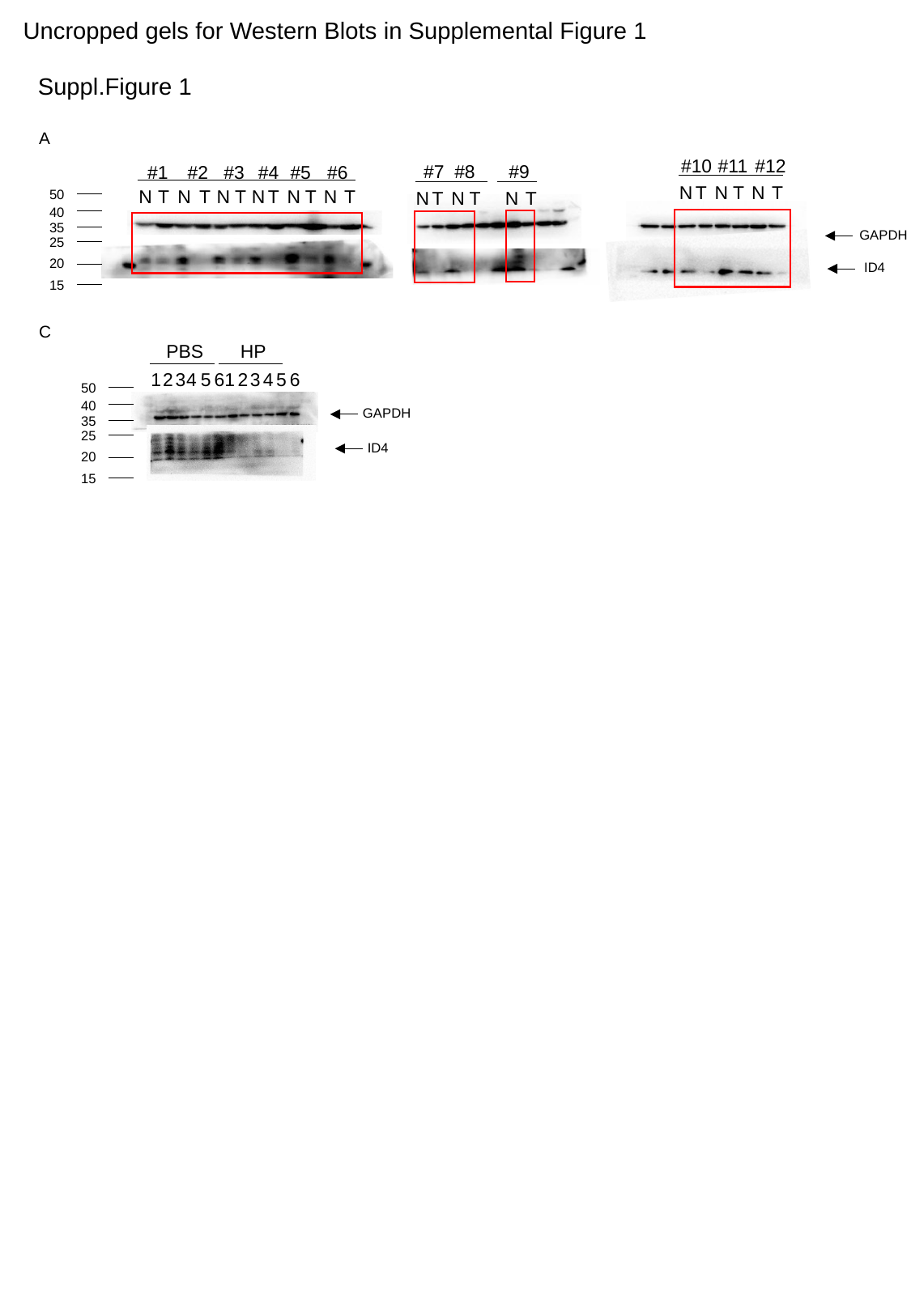

Uncropped gels for Western Blots in Supplemental Figure 1
Suppl.Figure 1
A
#10
#11
#12
#7
#8
#9
#1
#2
#3
#4
#5
#6
N
T
N
T
N
T
N
T
N
T
N
T
N
T
N
T
N
T
50
N
T
N
T
N
T
40
35
GAPDH
25
20
ID4
15
C
PBS
HP
1
2
3
4
5
6
1
2
3
4
5
6
50
40
GAPDH
35
25
ID4
20
15

## Slide 7
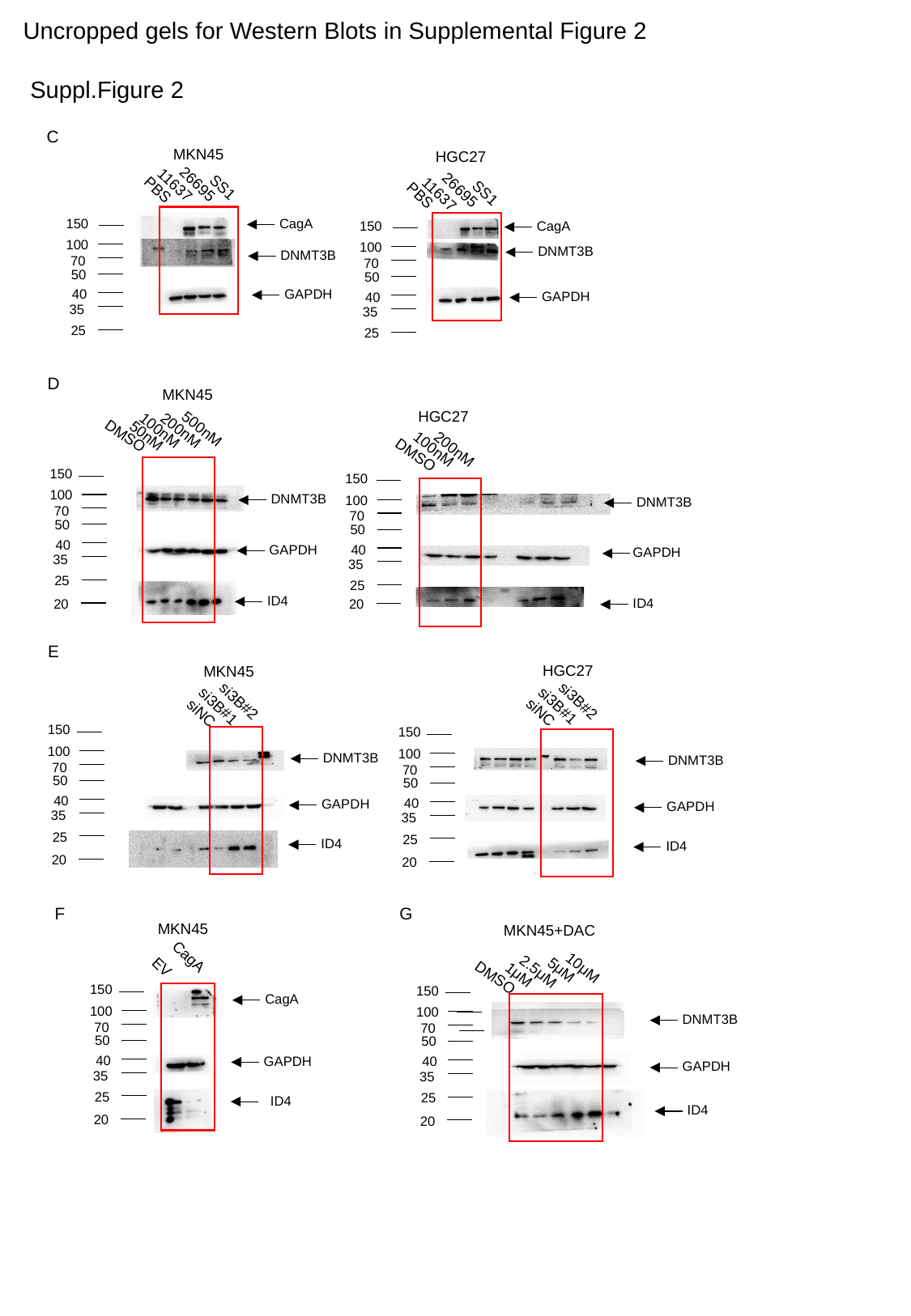

Uncropped gels for Western Blots in Supplemental Figure 2
Suppl.Figure 2
C
MKN45
HGC27
26695
11637
SS1
PBS
26695
SS1
PBS
11637
CagA
150
CagA
150
100
100
DNMT3B
DNMT3B
70
70
50
50
GAPDH
40
GAPDH
40
35
35
25
25
D
MKN45
HGC27
500nM
200nM
100nM
DMSO
50nM
200nM
100nM
DMSO
150
150
100
DNMT3B
100
DNMT3B
70
70
50
50
40
GAPDH
40
GAPDH
35
35
25
25
ID4
ID4
20
20
E
HGC27
MKN45
si3B#2
si3B#2
si3B#1
si3B#1
siNC
siNC
150
150
100
100
DNMT3B
DNMT3B
70
70
50
50
40
40
GAPDH
GAPDH
35
35
25
25
ID4
ID4
20
20
F
G
MKN45
MKN45+DAC
CagA
EV
10μM
2.5μM
5μM
DMSO
1μM
150
150
CagA
100
100
DNMT3B
70
70
50
50
40
GAPDH
40
GAPDH
35
35
25
25
ID4
ID4
20
20

## Slide 8
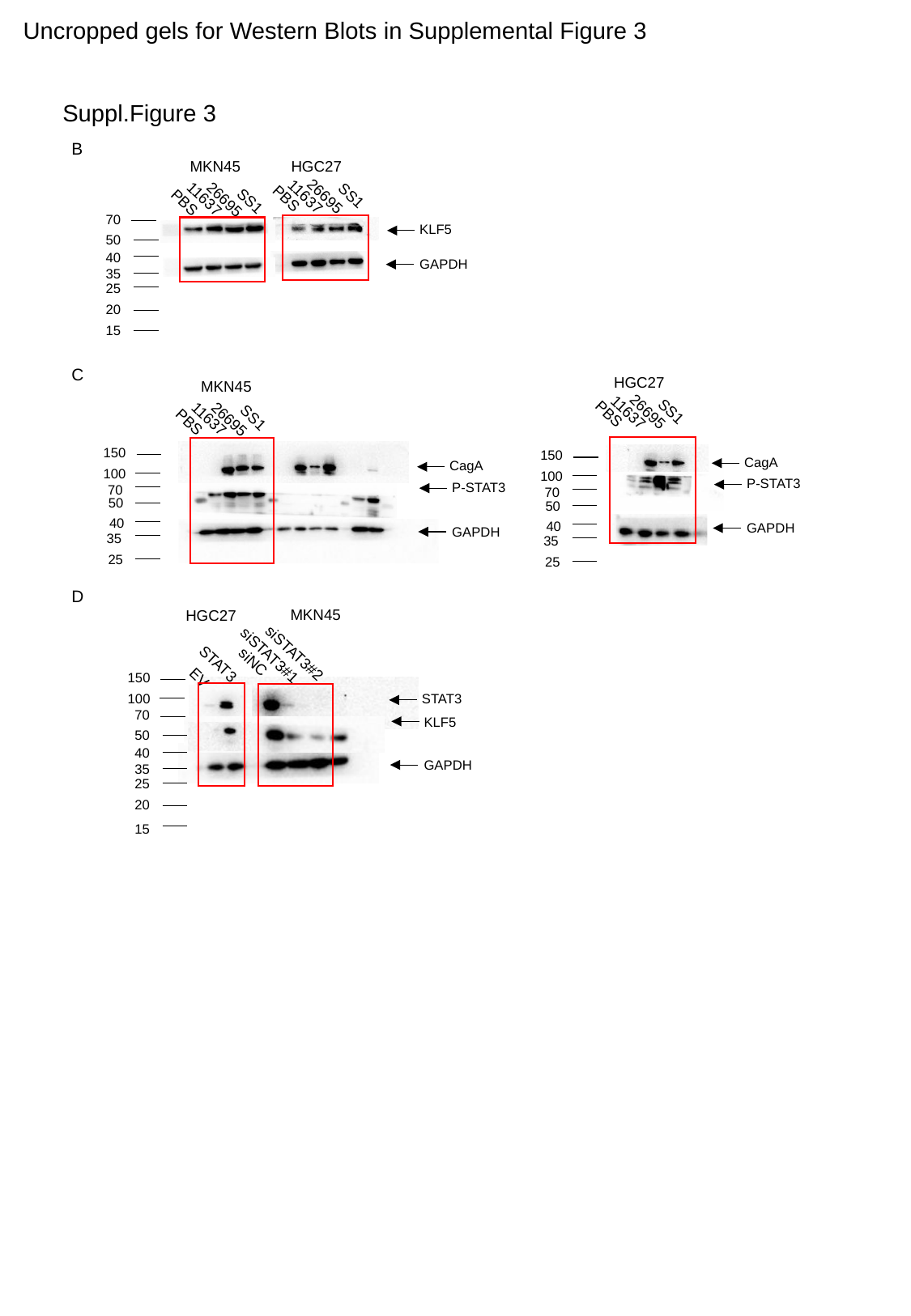

Uncropped gels for Western Blots in Supplemental Figure 3
Suppl.Figure 3
B
MKN45
HGC27
SS1
26695
PBS
11637
11637
26695
SS1
PBS
70
KLF5
50
40
GAPDH
35
25
20
15
C
HGC27
MKN45
SS1
26695
PBS
11637
SS1
11637
26695
PBS
150
150
CagA
CagA
100
100
P-STAT3
P-STAT3
70
70
50
50
40
40
GAPDH
GAPDH
35
35
25
25
D
MKN45
HGC27
siSTAT3#2
siSTAT3#1
STAT3
siNC
EV
150
STAT3
100
70
KLF5
50
40
GAPDH
35
25
20
15

## Slide 9
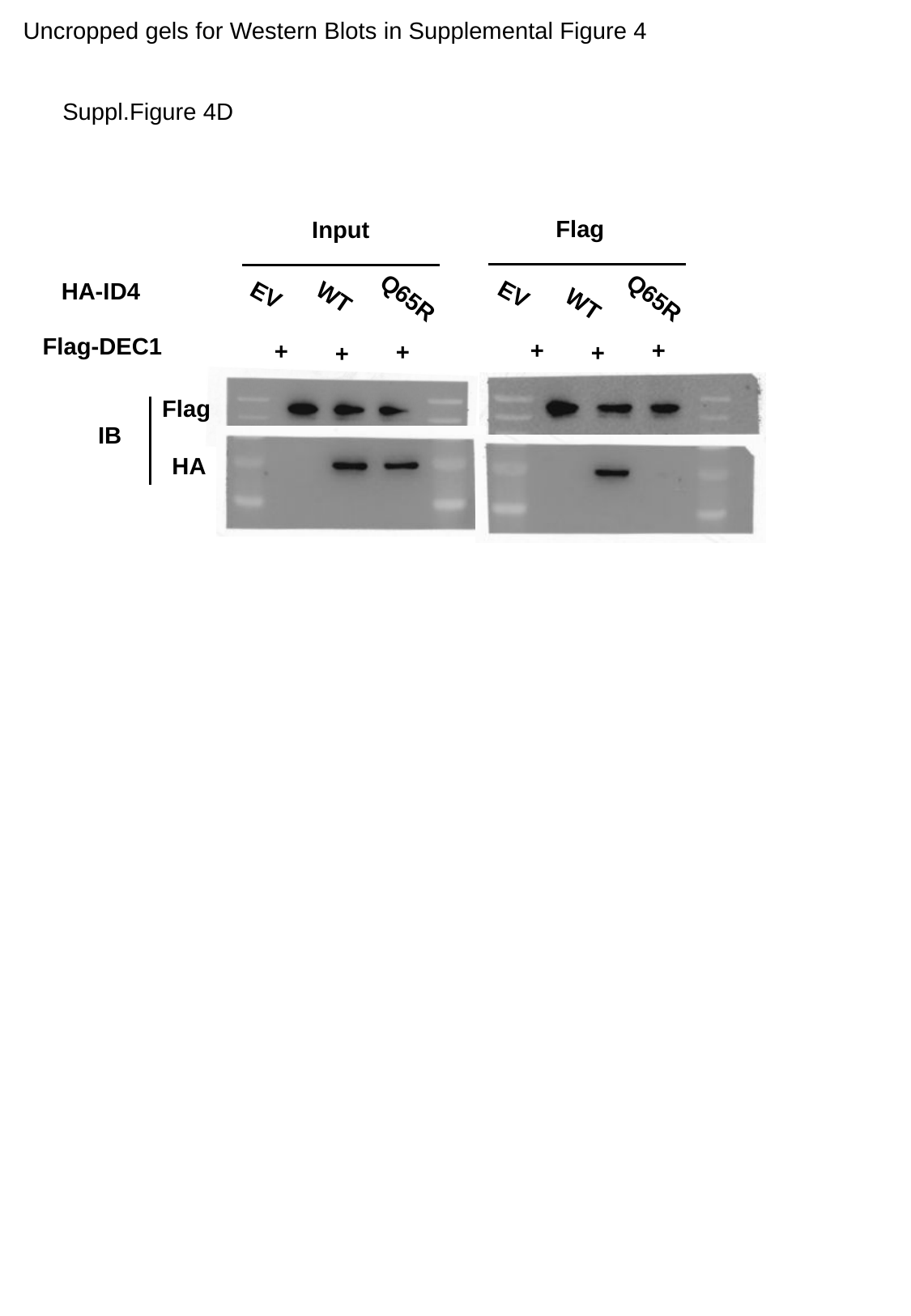

Uncropped gels for Western Blots in Supplemental Figure 4
Suppl.Figure 4D
Flag
Input
HA-ID4
EV
EV
WT
Q65R
Q65R
WT
Flag-DEC1
+
+
+
+
+
+
Flag
IB
HA

## Slide 10
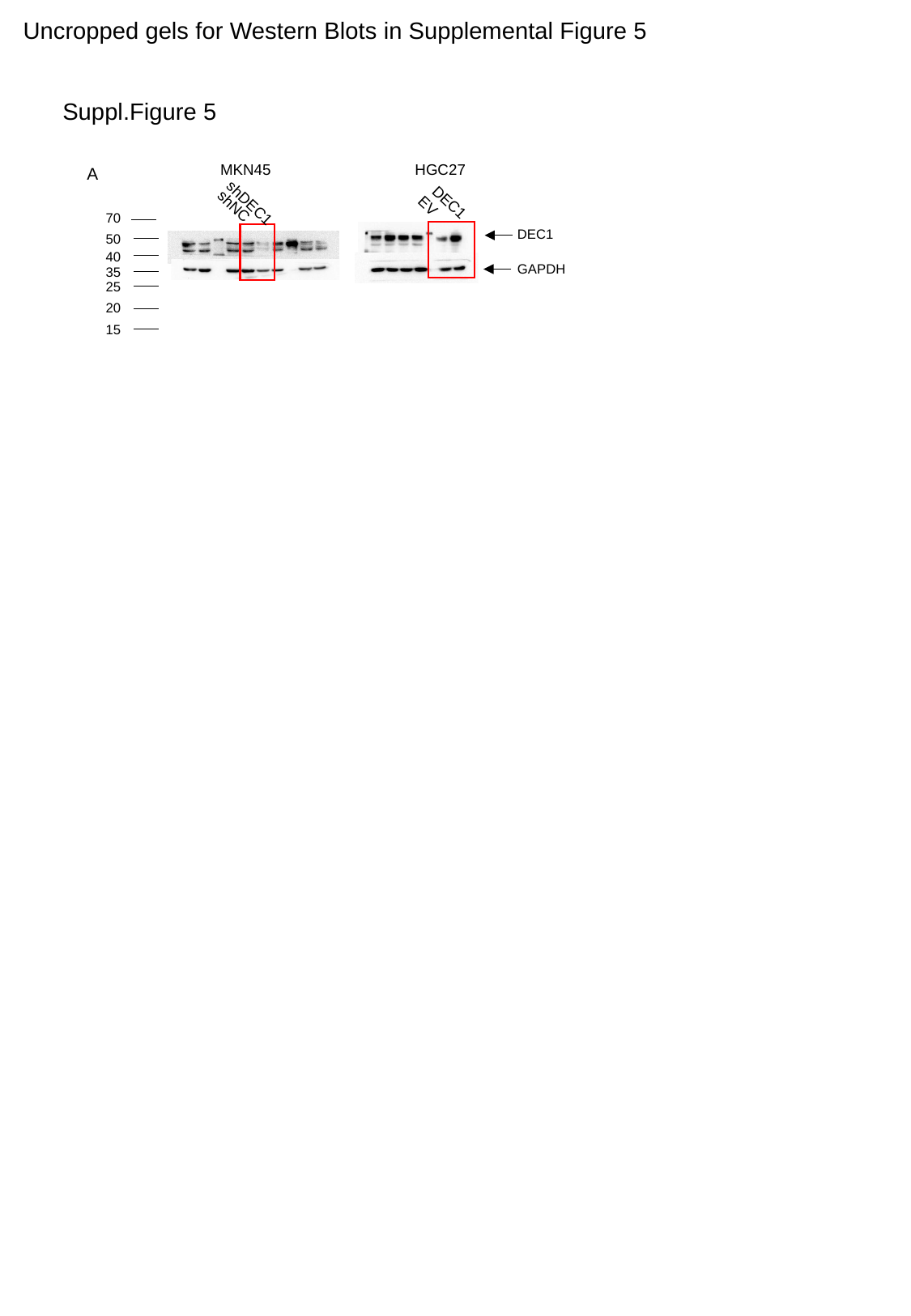

Uncropped gels for Western Blots in Supplemental Figure 5
Suppl.Figure 5
MKN45
HGC27
A
shDEC1
DEC1
EV
shNC
70
DEC1
50
40
GAPDH
35
25
20
15

## Slide 11
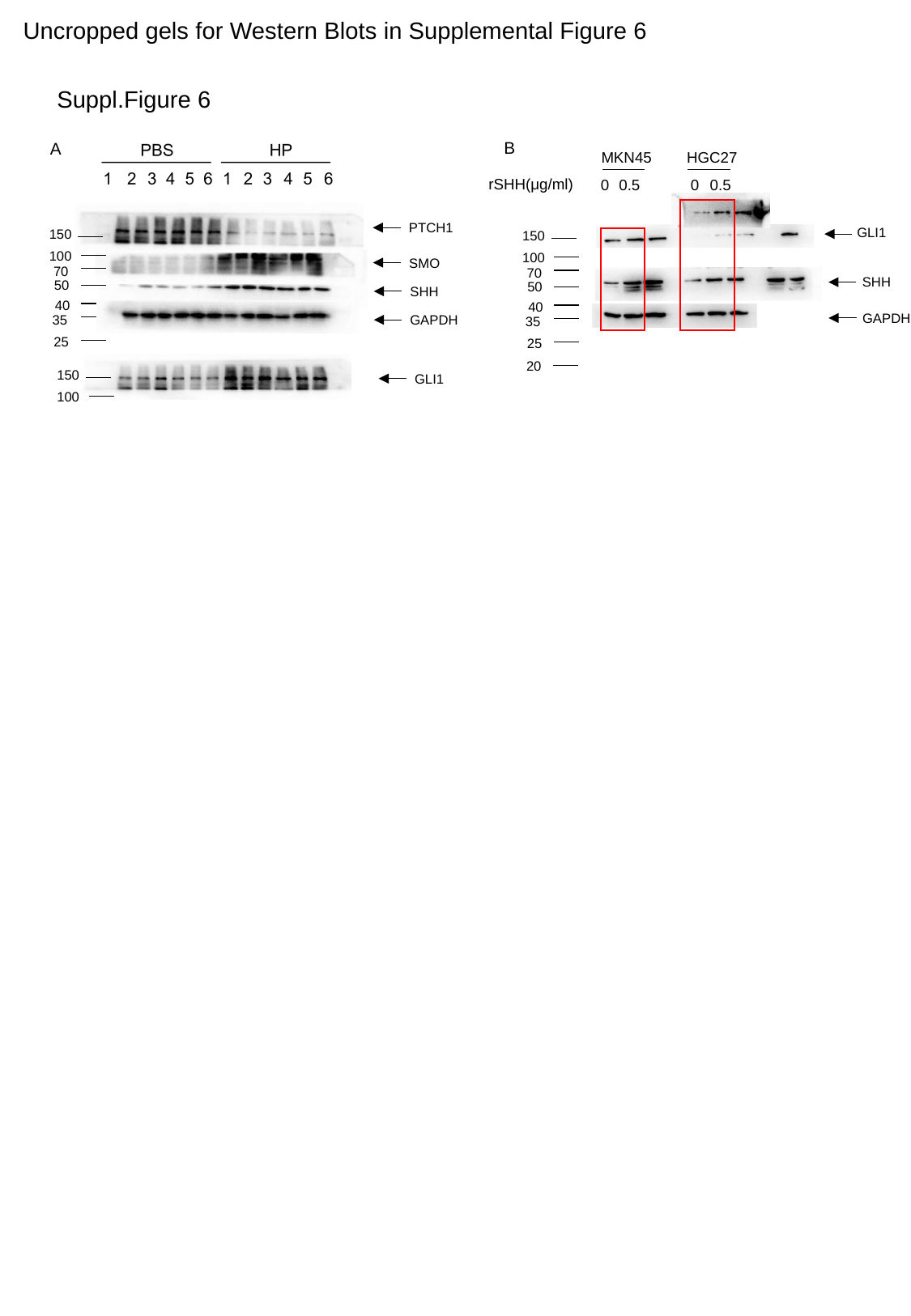

Uncropped gels for Western Blots in Supplemental Figure 6
Suppl.Figure 6
B
A
MKN45
HGC27
rSHH(μg/ml)
0
0.5
0.5
0
PTCH1
GLI1
150
150
100
100
SMO
70
70
SHH
50
50
SHH
40
40
GAPDH
35
GAPDH
35
25
25
20
150
GLI1
100

## Slide 12
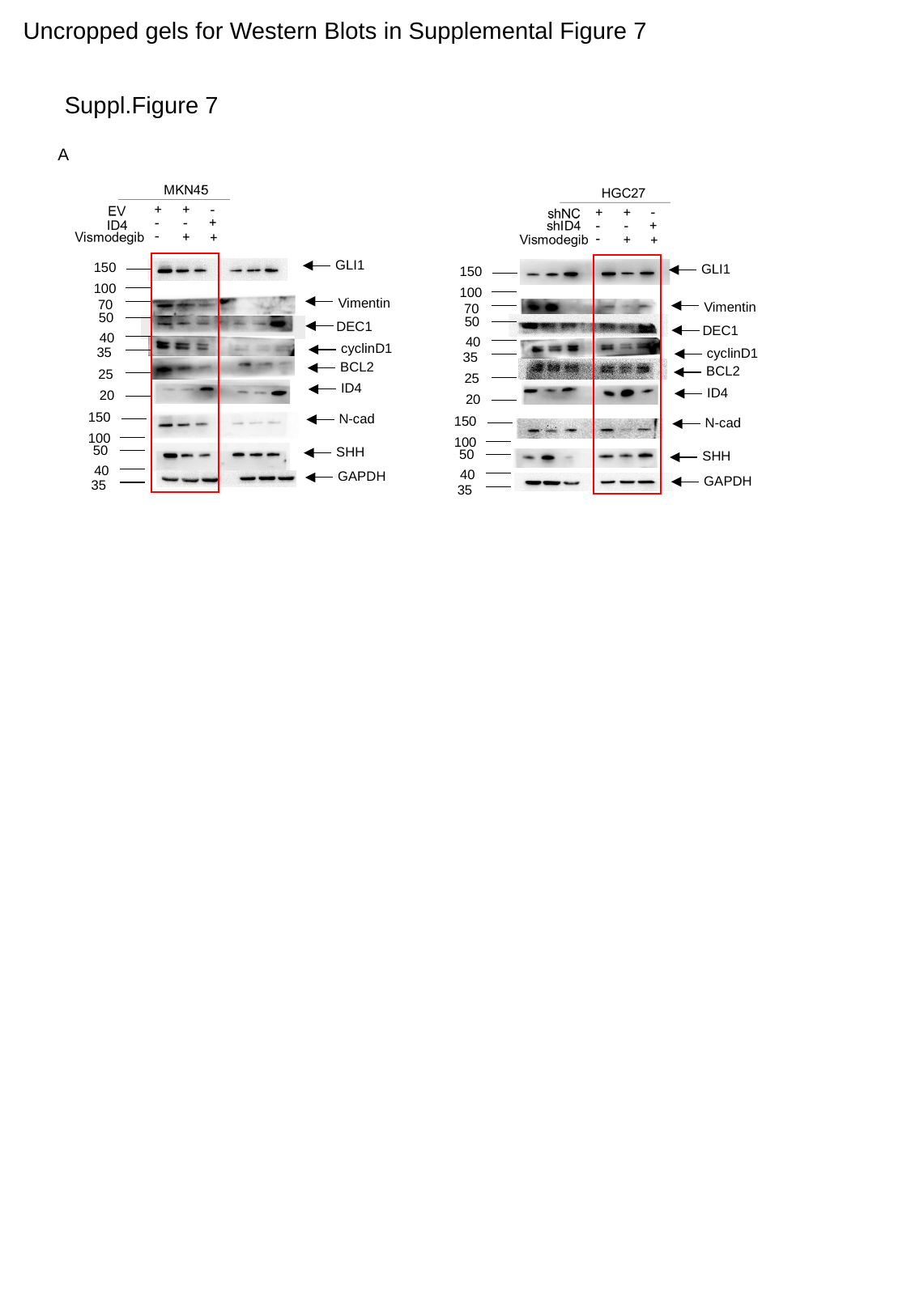

Uncropped gels for Western Blots in Supplemental Figure 7
Suppl.Figure 7
A
GLI1
150
GLI1
150
100
100
Vimentin
70
Vimentin
70
50
50
DEC1
DEC1
40
40
cyclinD1
35
cyclinD1
35
BCL2
BCL2
25
25
ID4
ID4
20
20
150
N-cad
150
N-cad
100
100
50
SHH
50
SHH
40
40
GAPDH
GAPDH
35
35
